# Ab Initio-Based Modeling of Thermodynamic Cyclic Voltammograms: A Benchmark Study on Ag(100) in Bromide Solutions

**DOI:** 10.1021/acs.jctc.3c00957

**Published:** 2023-12-01

**Authors:** Nicolas Bergmann, Nicolas G. Hörmann, Karsten Reuter

**Affiliations:** Fritz-Haber-Institut der Max-Planck-Gesellschaft, Faradayweg 4-6, D-14195 Berlin, Germany

## Abstract

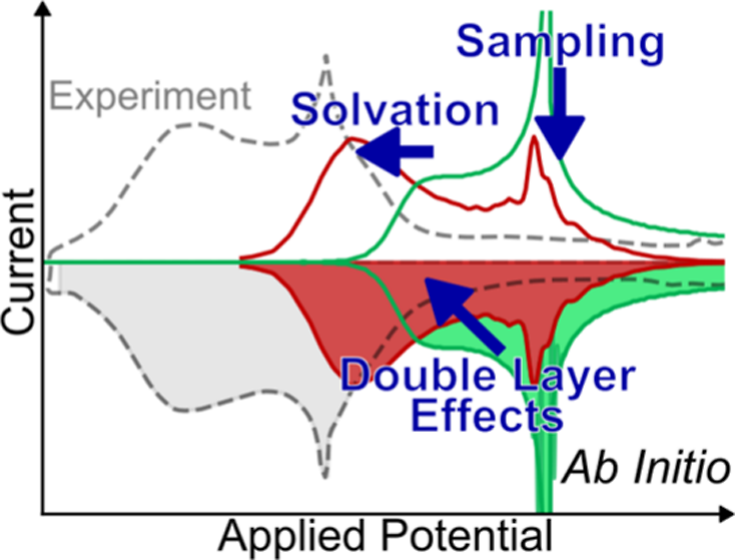

Experimental cyclic voltammograms (CVs) measured in the slow scan rate limit can be entirely described in terms of the thermodynamic equilibrium quantities of the electrified solid–liquid interface. They correspondingly serve as an important benchmark for the quality of first-principles calculations of interfacial thermodynamics. Here, we investigate the partially drastic approximations made presently in computationally efficient calculations for the well-defined showcase of an Ag(100) model electrode in Br-containing electrolytes, where the nontrivial part of the CV stems from the electrosorption of Br ions. We specifically study the entanglement of common approximations in the treatment of solvation and field effects, as well as in the way macroscopic averages of the two key quantities, namely, the potential-dependent adsorbate coverage and electrosorption valency, are derived from the first-principles energetics. We demonstrate that the combination of energetics obtained within an implicit solvation model and a perturbative second order account of capacitive double layer effects with a constant-potential grand-canonical Monte Carlo sampling of the adsorbate layer provides an accurate description of the experimental CV. However, our analysis also shows that error cancellation at lower levels of theory may equally lead to good descriptions even though key underlying physics such as the disorder–order transition of the Br adlayer at increasing coverages is inadequately treated.

## 1 Introduction

Cyclic voltammetry is a widely employed electrochemical experiment to characterize electrocatalytic processes occurring at electrified solid–liquid interfaces.^1,2^ A cyclic voltammogram (CV) records the electric current *j* observed while sweeping an applied electrode potential ϕ_E_ at a constant scan rate ν upward and downward within a given potential window.^3−6^ Peaks in the resulting voltammogram *j*(ϕ_E_) are then interpreted as fingerprints of occurring electrochemical reactions,^5^ whose fundamental nature can experimentally be uncovered by, e.g., investigating the CV’s dependencies on pH, applied potential limits, scan rate, or the electrolyte’s chemical composition.^7,8^ The derived assignments are often not unambiguous though and could strongly benefit from independent and predictive-quality computational modeling. Whenever diffusion limitations are absent, CV currents (*j* = dσ/d*t*) within the stable potential window of the electrolyte directly relate to changes in the equilibrium electronic surface charge dσ, due to differential electrode potential changes dϕ_E_ induced by the constant scan rate ν = dϕ_E_/d*t*. In this case, the dominant charging processes are the polarization of the electrolyte solution via double layer (DL) charging (dσ_DL_) and Faradaic processes, in which charged particles transfer across the electrode. In the defined case of a stable model electrode surface that neither reconstructs nor dissolves, on which we will focus here, the electronic charge transfer (dσ_a_) due to the latter processes stems entirely from electrosorption of adsorbates a onto the surface.^5,9,10^ dσ_a_ is then given by the change in adsorbate coverage θ_a_ multiplied by the number of exchanged electrons per adsorbate, aka the electrosorption valency *l*_a_.^11,12^ One can thus formally write
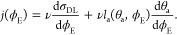
1With σ_DL_(ϕ_E_) often a quasi-constant baseline current, the theoretical modeling of a CV correspondingly requires an accurate description of the coverage versus potential relation θ_a_(ϕ_E_), as well as an appropriate consideration of the electrosorption valency *l*_a_(θ_a_, ϕ_E_). In the slow scan rate limit, both of these quantities are thermodynamic equilibrium quantities. In this respect, the corresponding experimental CVs also serve as important benchmarks for the quality of theoretical predictions of the interfacial thermodynamics.

It is with this motivation to benchmark various prevalent thermodynamic modeling choices and approximations that we here study the CV of a Ag(100) electrode in a Br^–^-containing electrolyte. This is a suitable and experimentally well-studied prototype system,^13−15^ for which high-quality CVs are available and in which electronic charge transfer arises from the electrosorption of Br^–^ ions onto defined high-symmetry sites of an otherwise rigid Ag(100) lattice. We specifically compare popular choices made in three significant modeling steps: the modeling of the liquid–solid interface, the determination of the energetics at applied potential conditions, and the statistical mechanics description are used to obtain macroscopic averages of *l*_a_ and θ_a_ from the atomistic energetics. The focus is thereby on computationally efficient approaches based on density-functional theory (DFT) calculations using a slab model for the electrode and without explicit representation of the electrolyte solution. We thus compare vacuum calculations to those in an implicit solvent environment, consider applied potential effects in first and second order,^16,17^ and apply mean-field and lattice-based grand-canonical Monte Carlo (GC-MC) sampling.^10,14^ The analysis shows that only higher order thermodynamics coupled to the lattice GC-MC sampling consistently recreates the characteristic peak shape and integral of the experimental CVs for the right reasons.

## 2 Experimental CVs of Ag(100) in Bromide Solutions

**Figure 1:**
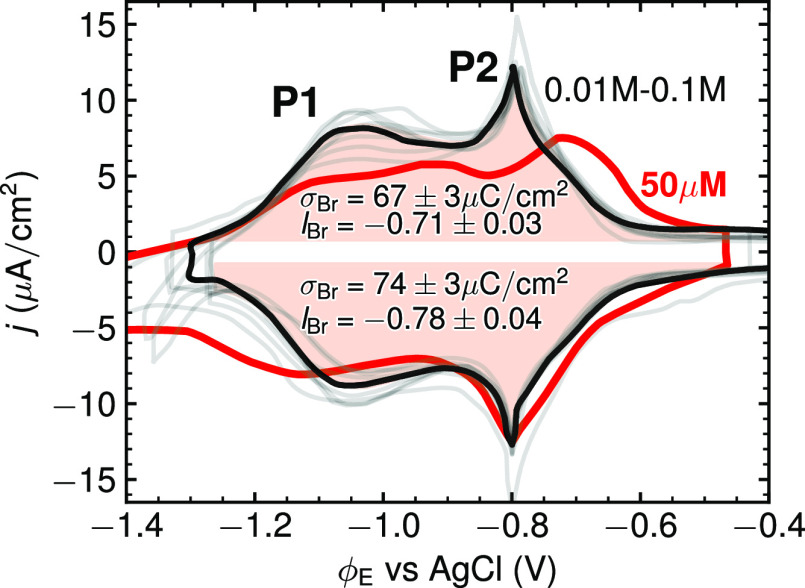
Ten experimental CVs of Ag(100) in Br^–^-containing electrolytes (gray lines) obtained from the echemdb database,^18^ with ν- and *c*_Br^–^_-normalized to 50 mV/s and 0.1 M, respectively. The CV measured by Nakamura et al.^15^ (bold black line) is henceforth taken as representative experimental reference in all figures below. In the anodic sweep direction, P1 marks the CV peak corresponding to the onset of Br electrosorption. At P2, the second order disorder–order phase transition occurs to the final *c*(2 × 2) Br-covered surface. Integrating the CV over the potential range from [−1.3 V, −0.4 V] vs AgCl (indicated by the red shaded area) and subtracting the capacitive DL baseline current contribution yields the total transferred electrosorption charge σ_Br_ and derived from it the electrosorption valency *l*_Br_ in the anodic and the cathodic sweep direction. The values quoted in the figure correspond to the average and standard deviation over the 10 CVs. The 10 CVs are from measurements at higher electrolyte concentrations (0.01–0.1 M), where thermodynamic CVs can be obtained at the applied scan rates. This is contrasted by the CV shown as a red line that was measured at *c*_Br^–^_ = 50 μM.^19^ In this curve, Br^–^ electrosorption’s kinetic limitations become visible through the peak hysteresis between the anodic and cathodic sweep directions. See the Supporting Information for the individual CVs and their references as well as all details regarding the CV normalization and integration.

The model system Ag(100) in Br^–^-containing electrolytes and its CV has been studied extensively.^13−15,20−24^Figure 1 shows a collection of digitized experimental CVs, using the echemdb database.^18^ The CVs span a range of electrolyte concentrations and cations. CVs with concentrations above 10 mM exhibit no significant hysteresis, i.e., the peak positions in the anodic sweep direction are essentially identical to those of the cathodic sweep direction, indicating the thermodynamic character of the experiments.^5^ Kinetic limitations, likely due to Br^–^ diffusion,^19^ only become relevant at much smaller concentrations (bold, red curve in Figure 1), which will not be studied here.

In general, the “butterfly”-shape of the CV is characterized by a first shoulder (peak P1) at lower potentials, which is ascribed to the formation of a disordered Br-adlayer with θ_Br_ ≤ 0.3 monolayer (ML), as evidenced in surface X-ray scattering experiments by Wandlowski et al.^13^ The prominent sharp peak (P2) at ≈0.38 ML, i.e., ∼75% of the limiting coverage 0.5 ML, marks the second order disorder–order phase transition where phase boundaries between different sublattices of Br adlayers are continuously removed,^25,26^ ultimately resulting in an ordered *c*(2 × 2) Br adlayer with 0.5 ML coverage at high potentials.^13,14,20^

The total transferred electronic charge σ_Br_, as determined by integrating the CV without baseline currents (red shaded area in Figure 1), indicates that the electrosorption valency is a noninteger. Assuming a nominal full electron transfer during electrosorption of Br^–^, i.e., *l*_Br_ = −1, the expected transferred electronic charge to a 0.5 ML adlayer would be σ_Br, nominal_ = 94 μC/cm^2^. This is 25% higher than the actually measured value of σ_Br_ ≈ 70 μC/cm^2^, cf. Figure 1. Additionally, ref (13) reported a non-Nernstian potential shift of the P2 peak of 110 meV per decadic logarithm of the Br^–^ concentration. Both of these observations are consistent with a noninteger electrosorption valency *l*_Br_ ∼ −0.75, where *l*_Br_ is defined as^11^
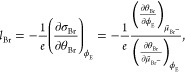
2where *e* is the elementary charge and  is the Br^–^ electrochemical potential. While not further discussed here, the known interdependencies of electrosorption valency and CV peak shapes^27^ indicate that this nonideal *l*_Br_ value might as well explain the cation-dependence of the peak shape and integral observed in ref (15).

## 3 Theory

**Figure 2:**
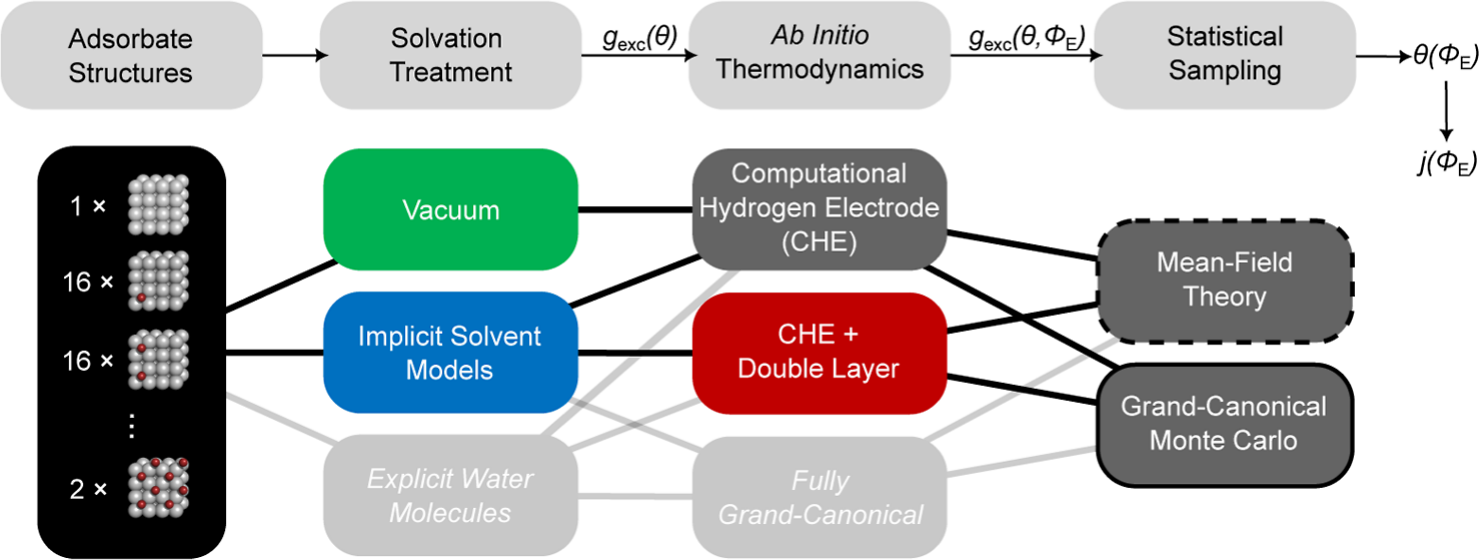
Typical modeling steps to derive thermodynamic CVs from first-principles calculations include the construction of a range of adsorbate structures on an electrode (shown in the left panel from the top view) in a chosen solvent environment, the assessment of their stability using an ab initio thermodynamics approach, and finally the determination of macroscopic averages via statistical sampling. The color codes and line forms of the different boxes reflect the colors used for corresponding data in all Figures below. While included in this schematic, we do not further consider simulations with explicit water molecules and with fully grand canonical ab initio thermodynamics due to their high computational cost.

While computing CVs with effective models, e.g., mean-field or lattice Hamiltonians, based on fitted experimental parameters has a long tradition,^14,23,28^ theoretical descriptions determined from first-principles methods based on DFT calculations are comparably recent.^9,16^ A typical simulation workflow for such ab initio thermodynamic CV modeling approaches is depicted in Figure 2. In the present work, we assess the impact of various choices at each of the modeling steps.

In the first step, we determine the DFT energetics of a range of adsorbate structures in a given solvent environment, considering prevalent approximations of the latter in the form of vacuum and an implicit solvent. Subsequently, we evaluate the relative stability of these structures as a function of electron and ion electrochemical potentials within an ab initio thermodynamics framework. Finally, we perform thermodynamic averaging to obtain the macroscopic averages of θ_Br_ and *l*_Br_. Using eq 1, this then yields the computed CV curve, where we disregard the essentially structureless baseline current contribution σ_DL_ that was also approximately removed from the experimental CVs.

### 3.1 Adsorbate Structures

Br is experimentally known to adsorb onto the 4-fold hollow sites of Ag(100) with a maximum coverage of 0.5 ML in a regular *c*(2 × 2) arrangement as consistent with high nearest-neighbor (NN) repulsions.^22^ We correspondingly construct a systematic data set of adsorbate structures by enumerating all configurations in a (4 × 4) Ag(100) surface unit-cell that do not exhibit NN occupations.^14^ In total, this yielded 28 symmetrically unique structures. To include information about the strong NN interactions in the data set, we add a single 9/16 ML structure with one additional Br on an empty site of the *c*(2 × 2) structure.

### 3.2 Computational Method and Solvation Treatment

Different levels of theory exist to model the solid–liquid interface.^29^ In principle, the most precise ab initio method uses explicit electrolyte and solvent molecules. These calculations can model effects such as hydrogen bonding networks toward surface adsorbates,^30^ potential-dependent solvent orientation, and concomitant capacitive responses,^31,32^ as well as electronic effects of the solid–liquid interface.^33,34^ However, the realistic treatment of the electrolyte solution requires significant computational resources, due to the necessity of performing molecular dynamic simulations, especially with the appropriate inclusion of the electrode potential.^30−32^

We therefore focus on a simpler, less-expensive model of a coarse-grained electrolyte: the so-called implicit solvent (IS). Here, instead of treating the electrolyte solution atomistically, it is described by a continuum model, which includes the dielectric and electrolyte screening.^29^ While this approach lowers the computational effort significantly, the use of IS requires parametrization of model aspects such as the solvation cavity and the dielectric response.^35^ For the IS, we rely on the SCCS model^36,37^ with solvent parameters from Hörmann et al.^10,38^ as implemented in the Quantum ENVIRON package.^36^

The energetics of all adsorbate structures is computed with DFT using the PBE functional^39^ to treat electronic exchange and correlation. All calculations are performed with the Quantum ESPRESSO package^37,40^ and ultrasoft pseudopotentials from the GBRV database (GBRV 1.5),^41^ and are managed with the AiiDA-Quantum ESPRESSO pw workflow.^42^ The (4 × 4) supercells employed to model the extended Ag(100) electrode comprise symmetric six layer slabs that are separated by a vacuum region of 18.5 Å. Keeping the two innermost slab layers frozen at the optimized bulk distance, all structures are fully relaxed to energy and force thresholds below 1.0 × 10^–4^ Ry and 5.0 × 10^–3^ Ry/bohr, respectively. Convergence tests indicate that at the employed computational settings (ecutwfc = 45 Ry, ecutrho = 360 Ry for the plane wave basis set, (4 × 4 × 1) gamma-centered *k*-point grid) the Br adsorption energies, *E*_ads_, are converged to within 0.01 eV, with further details on the DFT calculations provided in the Supporting Information.

With respect to the most relevant properties of the studied system, namely, the work function and the adsorption energies, previous work indicates a better performance of the PBE functional as compared to other semilocal functionals.^43,44^ Nonetheless, PBE is known to underestimate formation energies of bulk halides by ∼0.4 eV^45,46^ and similar errors are reported for the adsorption energies of according species.^44,47^ This generally needs to be kept in mind when judging predicted absolute CV peak positions, and we will return to this point below. Fortunately, this uncertainty does not directly affect the here first aspired relative comparison of different computational approaches to the CV modeling that we consistently all base on the same PBE energetics.

### 3.3 Ab Initio Thermodynamics

To evaluate the stability of adsorbate structures at applied electrode potential and experimental ion concentrations, we resort to two established electrochemical ab initio thermodynamics approaches.^16,27,48−51^

The most prominent method, the computational hydrogen electrode (CHE) approach,^16^ proved successful in replicating experimental CV peaks for a variety of systems.^9,52,53^ As shown in detail in ref (17), the CHE can be interpreted as a first order approximation of the fully grand canonical (FGC) energetics, only necessitating the energetics without electronic (and thus electrolyte) excess charges within the simulation cell, which we will simply refer to as potential of zero charge (PZC) conditions. Thus, the CHE can be evaluated in both vacuum and IS environments.^29,54−56^ Note, here and in previous works,^17^ the term CHE refers only to its common application at the so-defined PZC conditions, but not to its application at finite interfacial field or at finite, constant electronic excess charge.

In CHE, the stability of a structure α with *N*_Br_^α^ adsorbed Br atoms and *N*_sites_^α^ possible adsorption sites (and correspondingly a coverage θ_Br_^α^ = *N*_Br_^α^/*N*_sites_^α^) is given by the excess energy per surface site
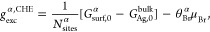
3with *G* here and henceforth referring to Gibbs free energies and the subscript 0 to an evaluation at the PZC. *G*_surf,0_^α^ (*G*_Ag,0_^bulk^) is correspondingly the Gibbs free energy of the surface structure α (Ag bulk), and μ_Br_ is the joint chemical potential for a charge-neutral Br species (Br = Br^–^ – *e*^–^) with
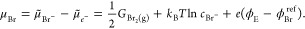
4Here,  is the Gibbs free energy of a Br_2_(g) gas-phase molecule, *c*_Br^–^_ is the ion concentration in mol/L, and ϕ_Br_^ref^ is the equilibrium potential for Br_2_(g) evolution at standard conditions. To reference the resulting DFT energies to the experimental Ag/AgCl reference electrode, we shift the values of ϕ_E_ using the literature experimental value of ϕ_E,ref_^Ag/AgCl^ = 4.637 V,^57^ i.e.,
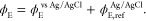
5

Simple substitution of the Gibbs free energy expressions above with DFT energetics (*G* → *E*) ignores vibrational zero-point and temperature effects, which can lead to sizable errors.^51^ To include these efficiently, we reexpress *g*_exc_^α,CHE^ as^10^
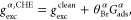
6where *g*_exc_^clean^ is the site-normalized cost of creating a clean interface
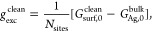
7and *G*_ads_^α^ is the coverage-normalized adsorption energy for a configuration α
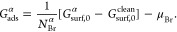
8

As vibrational free energy differences between metallic slabs and equally sized metallic bulk materials largely cancel, we can then approximate *g*_exc_^clean^ (eq 7) with differences in DFT energies. A similar reasoning applies to the energy differences in eq 8, which is why it is sufficient to only consider the vibrational modes of *N*_Br_^α^ adsorbates and of the Br_2_ gas-phase molecule to estimate *G*_ads_^α^ sufficiently accurate, see the Supporting Information for details of these vibrational calculations.

As the CHE approximation considers only charge-neutral surfaces (i.e., surfaces at their respective PZC), it intrinsically omits higher order potential dependencies of the interfacial energetics,^27,38^ thereby also a priori fixing the electrosorption valency to its nominal value, *l*_Br_ = −1. These higher order effects can be approximately included by using an efficient IS model, which allows charging the interfacial system. Explicitly evaluating the energetics at applied potential conditions^38^ then yields theoretical predictions for *l*_Br_.^27^ In fact, the CV current expression of eq 1 emerges naturally from such FGC energetics^10,27^ without adjustable parameters.

Further analysis of the FGC energetics shows that many of the aforementioned higher order dependencies in the potential are already captured by performing a second order Taylor expansion in the potential.^17^ The second order term can be interpreted as the energy contribution due to DL charging as well as due to geometric relaxation,^58,59^ which is not present in the CHE approximation.^10,38^ The expression of the free energy within this CHE+DL framework is^38^
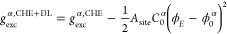
9where *A*_site_ is the adsorption site-normalized surface area of the substrate, *C*_0_^α^ is the capacitance, and ϕ_0_^α^ is the PZC of structure α (i.e., its work function).

In contrast to performing explicit calculations at each studied potential, the CHE+DL method allows one to capture the dominant potential dependencies in IS environments while necessitating only a few additional DFT evaluations at nonzero surface charge to obtain *C*_0_^α^.

While principally an approximation, in practice the second order CHE+DL method yields identical results as potential calculations in common IS models, where the electronic surface charges are varied explicitly.^17,58,59^ This is due to the very simple charge–potential relations within these IS models, which result in potential-independent interfacial capacitances.

Please see the Supporting Information for a complete listing of all DFT calculated thermodynamic properties, including *G*_ads_^α^ and corresponding *g*_exc_, the work functions ϕ_0_, and the implicit model determined *C*_0_^α^ as obtained from explicitly determining the charge-versus-potential relation.

### 3.4 Statistical Sampling

Knowing the thermodynamic stability of the set of adsorption structures α allows us to derive macroscopic observables by an appropriate statistical mechanics treatment that evaluates the configurational entropic contributions. Here, we follow two routes, namely, using the previously introduced approach based on mean-field theory (MFT)^10^ and an approach based on more rigorous lattice GC-MC sampling.^14,21,60^

Equilibrium coverages within MFT are determined via the construction of an approximate free energy landscape  as a function of θ_Br_ and subsequent minimization in θ_Br_-space. In previous work,^10^ we only considered a single, high-symmetry composition α at each coverage and determined  by interpolating  in θ_Br_ and adding an ideal-solution-like entropy term. Having sampled the full configuration space of the (4 × 4) supercell, we here construct  identically, but instead explicitly average over all configurations α at given θ_Br_ according to
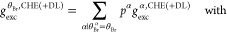
10
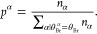
11Here, *n*_α_ is the statistical weight (multiplicity) of each symmetry-inequivalent structure α as determined by enumeration and symmetry reduction of all structures within the (4 × 4) cell. In the high-temperature and large-cell limit, this explicit average is consistent with the ideal-solution-like entropy term within MFT.^61^

In our GC-MC calculations, we map the adsorption patterns α on the 2D square lattice of the Ag(100) surface and fit the energetics *g*_exc_^α,CHE(+DL)^ under given conditions (ϕ_E_, *c*_Br^–^_) with a two-body cluster expansion (2b-CE), using the ICET python package,^62^ in a similar approach to ref (63). Next, we run the GC-MC simulations in a (18 × 18) 2D square lattice to obtain macroscopic averages for the coverage θ_Br_ under the respective conditions. More computational details and convergence tests are provided in the Supporting Information.

Finally, to derive CV currents from eq 1, we set *l*_Br_ = −1 for the CHE & MFT and CHE & GC-MC calculations, and use the analytic expression for *l*_Br_ from ref (10) for the CHE+DL and MFT analysis. For the CHE+DL & GC-MC analysis, we determine *l*_Br_ via eq 2 with differential coverage changes at points (ϕ_E_, *c*_Br^–^_) evaluated numerically by performing additional GC-MC simulations at slightly altered conditions (ϕ_*E*_ + dϕ_E_, *c*_Br^–^_ + d*c*_Br^–^_), cf. Supporting Information for details.

## 4 Results

In the subsequent sections, we assess the effectiveness of various modeling steps, as illustrated in Figure 2. Following the principle of Occam’s razor, we start from the most simple and computationally most efficient approach: vacuum-DFT calculations, CHE thermodynamics, and MFT statistical sampling. By improving the statistical sampling and gradually incorporating solvation and capacitive effects (at the level of an implicit solvent model), we carefully examine their influence on the overall outcomes, weighing their potential for improvement against the added complexity and cost that they introduce. We always employ the same scan rate and ion concentration as in the normalized experimental CVs of Figure 1, so that the results can be directly benchmarked against this reference.

### 4.1 Vacuum Energetics and CHE: Influence of the Statistical Sampling

#### 4.1.1 Robustness of the MFT Approach

We begin by comparing the performance of MFT and lattice GC-MC. As the influences of the statistical sampling method are largely independent of the ab initio thermodynamics modeling and the employed solvation model, we expect the resulting insights to then also transfer to the approaches incorporating the solvation and capacitive effects discussed below. Compared to the explicit GC-MC sampling, MFT seems more straightforward and computationally less demanding at first sight. However, as the MFT approach requires representing the coverage-dependent interfacial energetics *g*_exc_(θ_Br_) as a continuous function, it necessarily involves an interpolation of the discrete first-principles data available at the coverages that can be accessed in the employed finite-size surface unit-cell. Here, this is a (4 × 4) cell which correspondingly provides DFT energetic data at 1/16 ML coverage steps.

**Figure 3:**
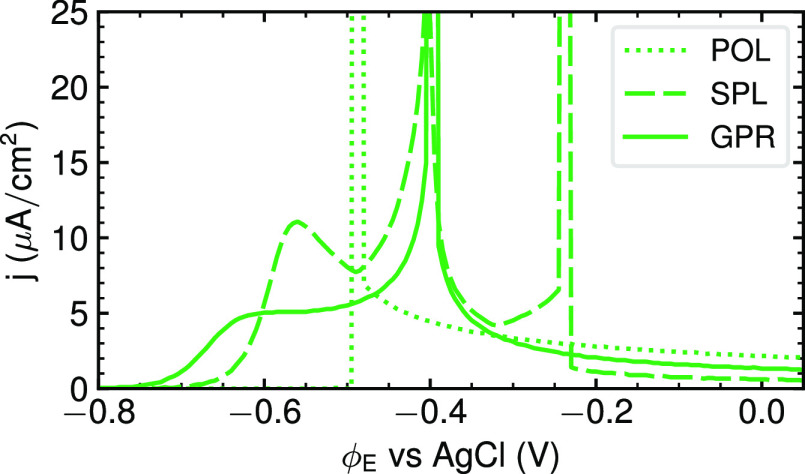
Effect of the employed interpolation method on CVs modeled with vacuum energetics, CHE, and MFT. POL = third order polynomial interpolation, SPL = cubic spline interpolation, and GPR = Gaussian process regression. Only the GPR interpolation recovers the double peak structure observed experimentally; cf. Figure 1.

Due to the strong, repulsive interactions between Br adsorbates, the interpolation method needs to be of a higher order than, for instance, the linear interpolations that have previously been employed for the modeling of CVs of H-electrosorption on Pt.^9,64,65^ To examine the sensitivity of the MFT approach on the employed interpolation method, we therefore compare third order polynomial (POL), Gaussian process regression (GPR, see the Supporting Information for more details), and cubic splines (SPL) interpolation. The resulting CVs are shown in Figure 3 and are discomfortingly different. While all three methods yield a CV centered around ∼−0.5 V versus AgCl, this CV has a widely differing shape consisting of one, two, and three subpeaks for the POL, GPR, and SPL interpolation, respectively.

This finding is easily explained as flexible interpolation methods can lead to nonconvex regions in the *g*_exc_(θ_Br_) function that result in discontinuous coverage changes as a function of the applied potential and thus sharp spikes in the predicted CV.^27^ The POL interpolation introduces no such region, while the more flexible methods GPR and SPL introduce one and two such regions, respectively. We are thus generally faced with the dilemma that a certain flexibility in the interpolation is required to appropriately capture the coverage dependence of *g*_exc_(θ_Br_), while too much flexibility can quickly lead to artifacts in the given finite DFT data. In principle, this may, of course, be remedied by increasing the θ_Br_-resolution of the DFT data. Yet, this would involve the use of larger surface unit-cells and more individual calculations, at concomitant strongly increased computational costs. At the present resolution, the GPR is the only method that recovers the experimentally observed double-peak structure of the CV, cf. Figure 1. We ascribe this to the controllable smoothness of the regressive properties of this method; see the Supporting Information for details, but note that the recovery of the experimental CV shape is only achieved after a careful tuning of the corresponding hyper-parameter. Even though the interpolation step is thus also critical for GPR interpolation, we focus on this method in the following.

**Figure 4:**
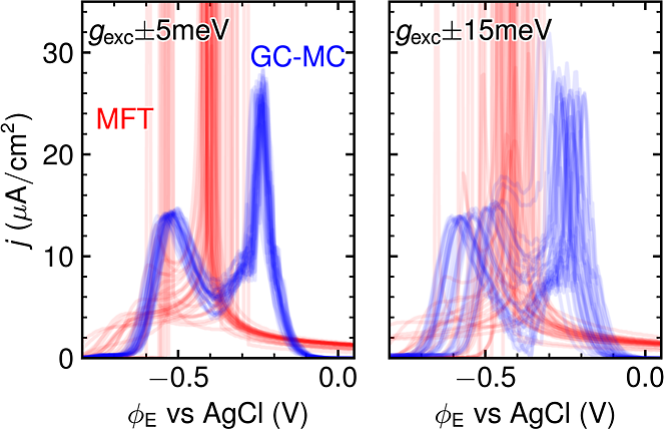
Sensitivity analysis of GPR-interpolated MFT- (red) and GC-MC (blue) CVs (using vacuum energetics and CHE) to white noise in the DFT data. Plotted are 30 CVs each, in which the underlying *g*_exc_(θ_Br_) DFT data were distorted by random errors in the range (left) ± 5 meV and (right) ± 15 meV.

A second issue for the interpolation method is its robustness to possible noise in the DFT data. Such noise can arise from multiple sources, ranging from not fully converged DFT calculations to finite ab initio molecular dynamics sampling in explicit solvation models.^66^Figure 4 shows corresponding CVs in which the underlying *G*_ads_^α^(θ_Br_) (i.e., the discrete data before interpolation) were distorted with white noise of varying strength (see the Supporting Information for more noise levels). Again a quite discomforting sensitivity is deduced in which already small noise levels induce strong shape changes dominated by spikes due to erratic coverage discontinuities. To put this into perspective, we also include in Figure 4 the CVs that are obtained when using the same distorted data as the basis for a GC-MC sampling. Specifically, we here used a 2b-CE with the interaction cutoff set to 4.3 Å, such that the expansion includes on-site energy and first and second NN interactions that are parametrized with the DFT data; cf. Supporting Information for more details on and convergence of the 2b-CE. With the exception of overall CV shifts, the GC-MC CVs retain their peak shape much better under the influence of noise, in fact even up to the high noise level shown in Figure 4.

The superior stability of GC-MC likely results from the fact that the noise can only affect the interaction weights of the GC-MC’s predetermined Hamiltonian, while it can alter the overall nature of the MFT Hamiltonian. In the present short-range 2b-CE, a change in the adsorption energy shifts only the entire CV peak. The first NN interaction is energetically so unfavorable that any small changes do not affect the essential blocking of NN occupations in the adsorbate lattice. As a result, actual variations in the peak shapes are only introduced by noise-induced variations in the weaker repulsive second NN interaction, where increasing or decreasing values merely stretch or compress the CV, cf. Supporting Information. This limited mapping induces an inherent robustness to errors. To be fair, one should note though that this is gradually lost when increasing the 2b interaction cutoff or including many-body interactions into the CE. As shown in the Supporting Information, we then also obtain somewhat larger distortions of the GC-MC CVs. However, they are never as large as those of the MFT CVs for the same noise level, and we also observe a systematic and rapid convergence of the simulated CVs with respect to an increase in the 2b interaction cutoff. This demonstrates that the robust short-range CE with only first and second NN interactions (interaction cutoff set to 4.3 Å) as in Figure 4 is fully sufficient for the present system and used henceforth as default.

#### 4.1.2 MFT vs GC-MC Sampling

**Figure 5:**
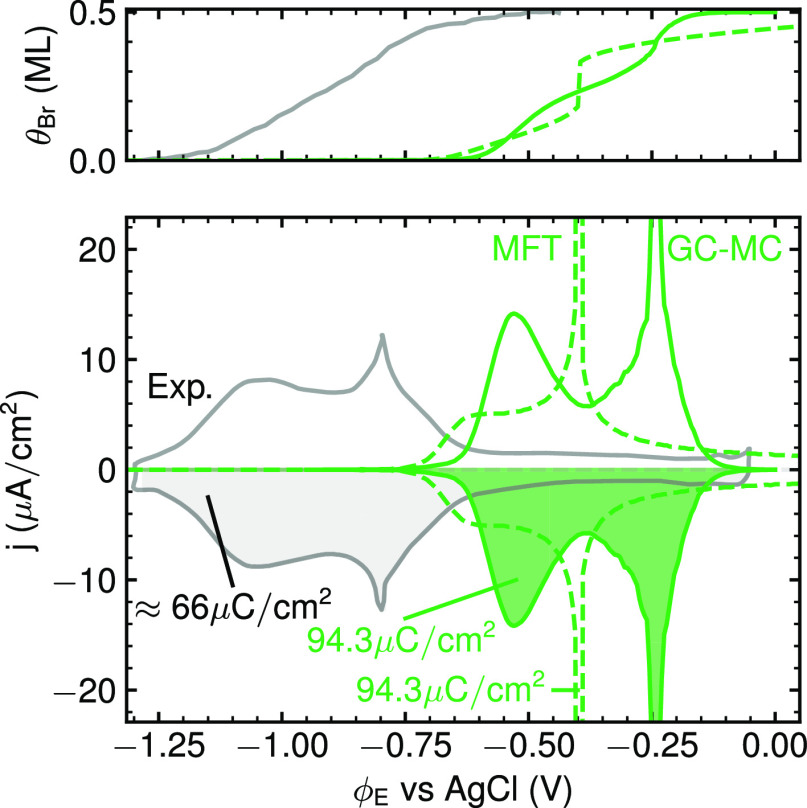
Comparison of the best-practice GPR-interpolated MFT (dashed, green line) and GC-MC (solid green line) CV with the normalized experimental CV from Figure 1 (solid, gray line).^15^ Both theoretical CVs are based on vacuum energetics and CHE. Also indicated is the total transferred electronic charge obtained from integrating each CV. The top panel shows the corresponding surface coverage. The experimental coverage isotherm is taken from chronocoulometry measurements from Wandlowski et al.^13^

The results of the last subsection reveal that while MFT is an easy and quick approach, its sensitivity to the employed interpolation method and to noise in the DFT data render it nonideal to model CVs with complex peak shapes. This assessment does thereby not even extend to its approximate handling of the configurational entropy. We assess the latter in Figure 5 where we directly benchmark the CVs obtained with the determined best-practice MFT and GC-MC model against the normalized experimental data. Both theoretical CVs are strongly shifted and more compressed compared to the experimental reference. In both methods, the onset of Br electrosorption occurs at ∼−0.6 V versus AgCl and is followed by a shoulder feature consistent with the experimentally observed peak P1 as discussed in Section 2. Similarly, both methods yield a sharper second peak P2 at higher potentials (MFT at −0.4 V vs AgCl, GC-MC at −0.25 V vs AgCl).

In detail, however, the two methods do predict quite different CV shapes, with the MFT approach with its nominally inferior sampling in fact somewhat better reproducing the experimental shape, in terms of both the more humplike character of the P1 peak and the sharp spikelike character of the P2 peak. Yet, with respect to the latter, one can clearly show that this is completely fortuitous. In the GC-MC simulations, the P2 peak arises as expected from a second order disorder–order phase transition of the Br adlayer. Using order parameters appropriate for (2 × 2) ordering,^67,68^ the freezing out of the ordered *c*(2 × 2) structure from a previously disordered lattice gas at potentials around P2 can nicely be discerned as shown in the Supporting Information. In fact, the employed short-range CE truncated to first and second NN interactions directly connects to a bulk of work with corresponding model Hamiltonians on square lattices. From such work, the nature of the disorder–order phase transition is well-known. For a site-blocking first NN repulsive interaction, the transition occurs at about 75–80% of the limiting coverage of 0.5 ML.^14,21,25,26,69^ Furthermore, this critical coverage θ_c_ varies only slightly in the presence of longer-range interactions and remains at 80% for a large range of repulsive second NN interaction energies.^69^ Fully consistent with this, peak P2 arises at θ_c_ ≈ 80% in our GC-MC simulations, and the previously discussed robustness of the simulation results in particular with respect to the P2 part of the CV directly correlates with the known robust and universal nature of this phase transition.

In contrast, MFT is by construction completely agnostic to such disorder–order physics. Here, the P2 peak derives simply from a discontinuous jump in θ_Br_ occurring between 0.20 and 0.35 ML, i.e., at 50–70% of the maximum coverage. As already stated, this jump is the result of a nonconvex coverage-dependence of *g*_exc_, and thus depends sensitively on the details of the DFT data points and the interpolation method. The good agreement of the MFT P2 peak shape is thus a prime example of right for wrong reasons, and we will see next that the worse prediction obtained for the superior GC-MC sampling is in fact the consequence of the hitherto still lacking treatment of solvation and capacitive effects.

### 4.2 GC-MC & CHE: Solvent Stabilization

**Figure 6:**
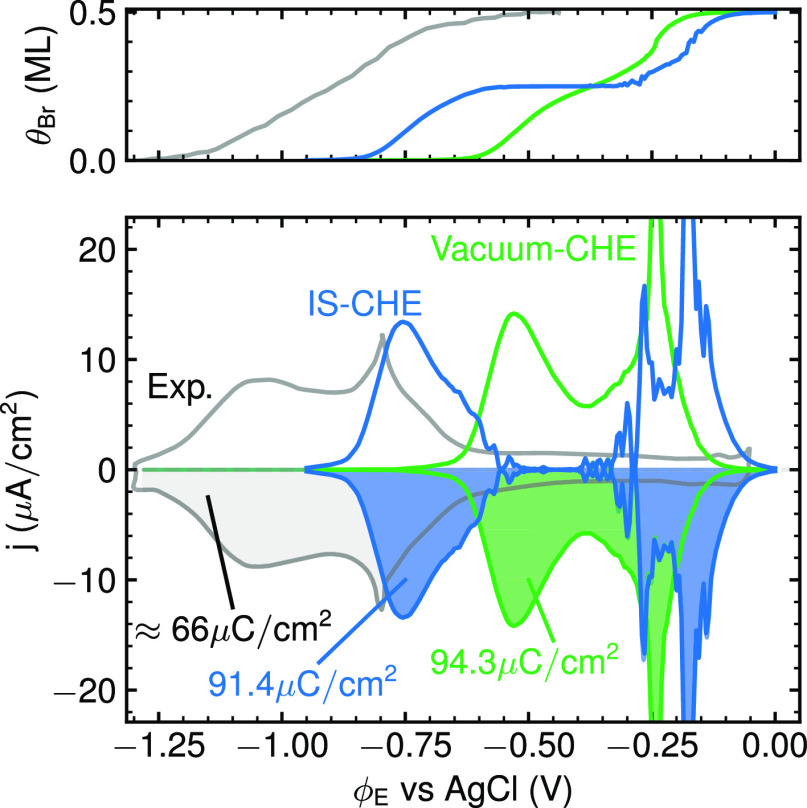
Same as Figure 5, but now comparing GC-MC and CHE CVs based on vacuum energetics (solid, green line) and implicit solvation (IS) energetics (solid, blue line) with the normalized experimental CV from Figure 1 (solid, gray line).^15^ Also indicated is the total transferred electronic charge obtained from integrating each CV. The top panel shows the corresponding surface coverage. The experimental coverage isotherm is taken from chronocoulometry measurements from Wandlowski et al.^13^

In view of the inherent deficiencies of the MFT sampling, we concentrated our ensuing analysis on GC-MC sampling. Apart from the differences in the overall CV shape with respect to the experimental reference, a second discrepancy of the aforementioned GC-MC CV obtained with vacuum energetics and the CHE was an overall offset by ≈0.5 V. Such a shift to more anodic potentials might well be due to the lack of solvent stabilization in the hitherto employed vacuum energetics. In our next analysis step, we correspondingly still stay within the CHE, but now employ the DFT energetics obtained with the implicit solvation model. Figure 6 compares the corresponding CV with that obtained with vacuum energetics and the experimental reference. Indeed, the onset of the implicit-solvent CV shifts to lower potentials, reflecting a stabilization of the respective low-coverage adsorbate configurations by the solvent model. However, this is accompanied by an opposite slight upward shift of the higher-coverage P2-peak part of the CV. As a result, the overall CV becomes much broader than the experimental reference, and de facto separates into two parts.

**Figure 7:**
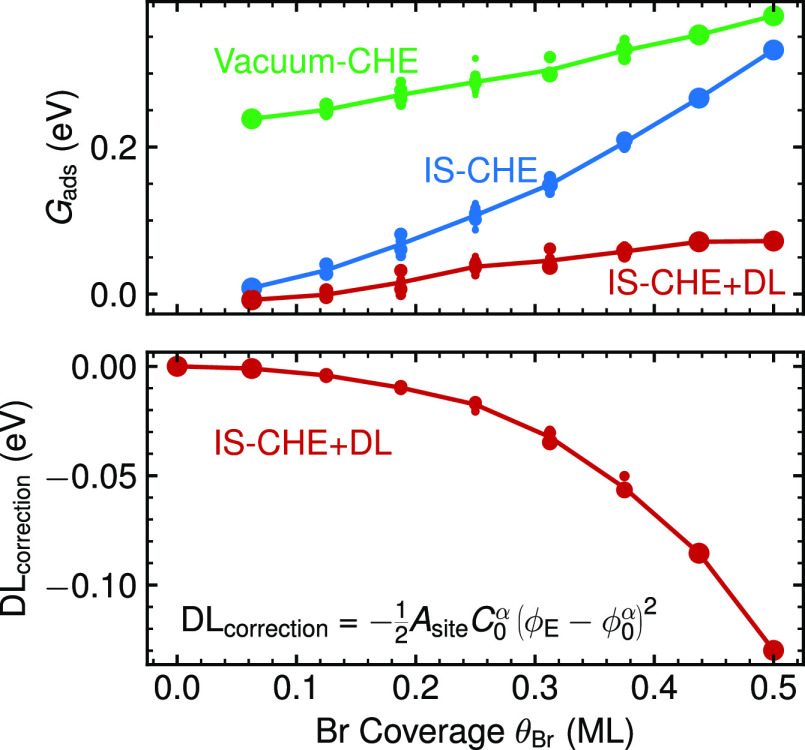
Adsorption energy *G*_ads_ for all DFT-calculated configurations α plotted against their respective Br coverage θ_Br_, evaluated at ϕ_E_ = −0.8 V vs AgCl (top panel), the center of the experimental CV. The size of the scatter points corresponds to *p*^α^ (eq 11). We show CHE values for vacuum (green) and implicit solvent energetics (blue) as well as the CHE+DL values within the implicit solvent model (red). The correction term introduced by the CHE+DL scheme (DL_correction_) is shown in the bottom panel. In both figures, the lines correspond to the weighted average values for each unique coverage.

**Figure 8:**
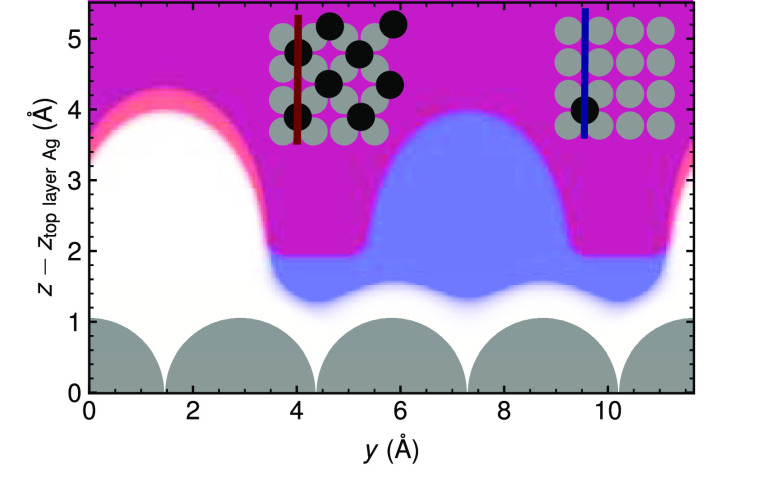
Side view of the solvation cavity of the implicit solvation model for a low-coverage *p*(4 × 4) (blue) and a high-coverage *c*(2 × 2) (red) Br adsorbate layer. The insets explain the position of the vertical cut above the surface. In the case of a low-coverage adsorbate layer, the dielectric medium extends much closer to the surface between the adsorbates, thus enabling higher solvent stabilization due to screening.

A direct comparison of the coverage-dependent adsorption energies in a vacuum and IS in the top panel in Figure 7 points to the origin of this separation. While *G*_ads_^CHE^(θ_Br_) at low coverages are stabilized by the solvent model by ∼250 meV per Br adsorbate relative to the vacuum energetics, the IS-induced stabilization diminishes with increasing coverage, becoming negligible at the highest coverage of θ_Br_ = 0.5 ML. In the short-range 2b-CE, this translates to a decrease in the onsite term of 203 meV and an doubling of the repulsive second NN interaction term from 60 meV in vacuum to 135 meV in implicit solvation. Overall, this then spreads the coverage isotherm as seen in Figure 6 and concomitantly the CV. The diminishing stabilization in turn is a direct consequence of the implicit solvent representation in the form of a dielectric continuum beyond a solvation cavity defined by a threshold electron density.^36^ As apparent from Figure 8, at low coverage this cavity extends to close to the surface in the large clean parts of the surface between the dilute Br adsorbates. In contrast, this is no longer possible at the small spacing between the Br adsorbates at the highest coverage. The stabilization in the IS model results from a simple screening of the repulsive electrostatic interactions between the Br adsorbates by the dielectric medium. With this medium being able to encapsulate the Br adsorbates much better at low coverages, a higher stabilization consequently arises as compared to the high-coverage case where this is no longer possible (as the solvent cannot penetrate between the adsorbates anymore). Even though the IS model is a coarse representation of the true solvation environment, this varying screening and concomitantly differing degrees of solvent stabilization should in principle be the correct physics. As in the case with the sampling before, we thus again arrive at the result that a nominally better modeling does not directly lead to an improved CV observable.

### 4.3 GC-MC & CHE+DL: Capacitive Charging Effects

**Figure 9:**
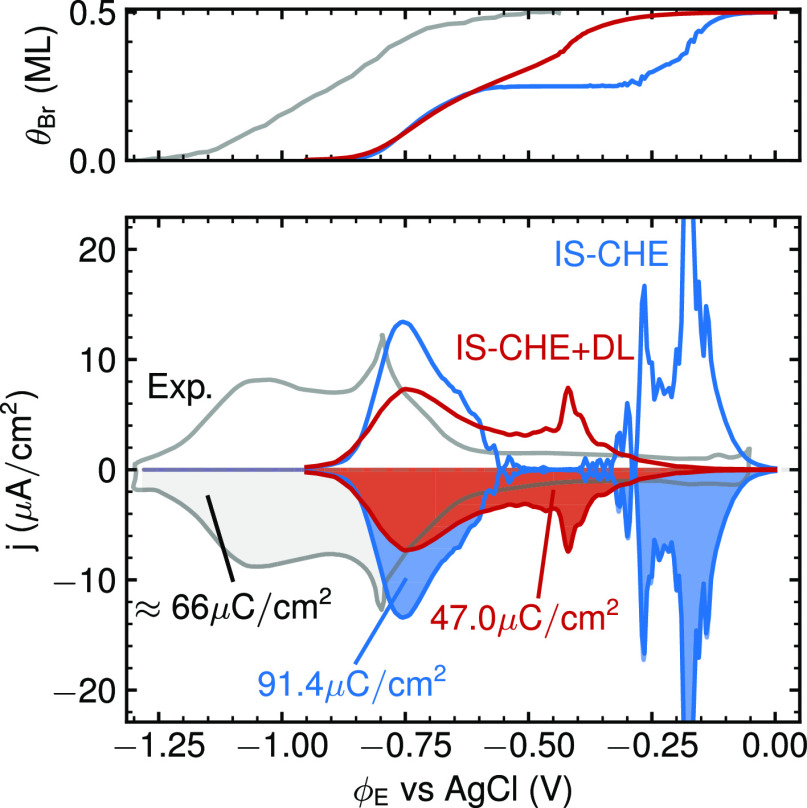
Same as Figures 5 and 6, but now comparing GC-MC & implicit solvation CVs based on CHE (solid, blue line) and implicit solvation energetics (solid, red line) with the normalized experimental CV from Figure 1 (solid, gray line).^15^ Also indicated is the total transferred electronic charge obtained from integrating each CV. The top panel shows the corresponding surface coverage. The experimental coverage isotherm is taken from chronocoulometry measurements from Wandlowski et al.^13^

The last missing piece in the modeling hierarchy is the consideration of capacitive charging effects via the CHE+DL approach. Figure 9 correspondingly compares the simulated GC-MC CV based on implicit solvation energetics at the CHE and CHE+DL levels with the experimental reference. Remarkably, the second order inclusion of the electrode potential largely reverts the excessive CV broadening observed previously when switching from vacuum to implicit solvation energetics at the CHE level, while at the same time, leaving the onset potential of the CV unchanged. As a result, a CV shape highly reminiscent of the experimental CV is again obtained, but with the entire CV now also located at more cathodic potentials closer to this reference.

This result can be rationalized by analyzing the quadratic DL correction term  that is introduced at this level of theory. Figure 7 shows the coverage dependence of this term when approximately evaluating it for ϕ_E_ = −0.8 V vs AgCl and thus at a potential that roughly corresponds to the center of the experimental CV. At such relevant potentials, the term becomes increasingly negative with increasing coverage and therefore effectively cancels the increased positive slope of the θ_Br_*G*_ads_^CHE^ CHE-term upon changing to implicit solvation energetics, cf. Figure 7. As shown in Figure 7, this leads to *G*_ads_^IS-CHE+DL^ exhibiting almost the same slope as that of *G*_ads_^vacuum-CHE^. In other words, the fortuitous agreement of the shape of the CHE plus vacuum energetics CV with experiment was the result of a cancellation of errors introduced by the simultaneously missing solvation and capacitive charging effects.

However, the CHE+DL approach not only improves the overall peak shape and absolute position of the CV. It also significantly reduces the total transferred electronic charge σ_Br_, i.e., the integrated area under the CV, as well as changes the relative height of the P1 and P2 peaks. Both of these changes again improve the comparison to the experimental reference. In particular, σ_Br_ was consistently overestimated within all previous modeling approaches, cf. Figures 5, 6, and 9, and is now in much better agreement with the experiment. Both of these effects arise from the electrosorption valency *l*_Br_(θ_Br_, ϕ_E_) that scales the overall CV, cf. eq 1, and that in the CHE+DL approach can now take values less negative than the nominal charge of −1.^38^ As shown in the Supporting Information, the CHE+DL *l*_Br_ is in fact not constant, but increases almost linearly from −0.7 to −0.45 over the potential window (aka coverage) of the CV and falls thus into the range estimated for the electrosorption valency from the experimental data, cf. Section 2. This potential dependence of *l*_Br_ then alters the relative heights of the P1 and P2 peaks, as less charge is transmitted per adsorbate at lower than at higher coverages. It is also only this noninteger value of *l*_Br_ that leads to the non-Nernstian potential shift of the P2 peak with Br^–^ concentration reported experimentally.^13^

Overall and gratifyingly, it is thus indeed the CV modeled at the nominally best level of theory that achieves the best agreement with the experimental reference, i.e., a CV obtained by GC-MC sampling, energetics accounting for solvation effects at least at the level of an implicit solvation model, as well as considering capacitive charging effects to the second order. In fact, considering that we have focused only on computationally efficient approaches that in many respects are still effective, prominently the description of the solvation environment by a mere dielectric continuum, this agreement down to width, shape, and integrated area of the CV is quite impressive. What remains as the largest discrepancy is the overall potential shift of about ∼0.3 V of the predicted CV versus the experimental data. We ascribe much of this difference to the employed semilocal PBE DFT functional and support this assignment with a recalculation of all vacuum DFT energetics with the revPBE functional, cf. Supporting Information for details. We obtain *G*_ads_ for all configurations α that are predominantly shifted by about ∼+0.15 eV as compared to the corresponding PBE values. Consequently, while the on-site term of a short-range 2b-CE based on these energetics becomes less stable by ∼0.15 eV, the first and second NN interactions remain unchanged. Obviously, the entire analysis of the last sections would thus hold in an analogous way for this CE, just with the entire simulated CVs shifted by ∼0.15 V to more cathodic potentials and thus even further away from the experimental reference. This agrees with the general expectation of an even weaker binding at the revPBE level and the knowledge that already the PBE underestimates the binding of halides.^44−47^ Of course, just testing one other semilocal functional does not do justice to the wealth of approximate DFT energetics that can in principle be obtained. Nevertheless, we believe that the provided singular example illustrates that this uncertainty in the energetics may prominently lead to overall shifts of the simulated CV. As such, the approximate DFT energetics are in our view the most likely candidate to explain the remaining discrepancy of the GC-MC CHE+DL CV based on implicit solvation energetics with respect to the experimental reference.

## 5 Summary and Conclusions

In this benchmark study, we have systematically analyzed prominent choices in the simulation workflow for thermodynamic CVs using Br electrosorption at a model Ag(100) electrode as a representative showcase. Focusing on computationally efficient, prevalent approaches, we analyzed the influence of an approximate account of the solvation environment in form of energetics calculated within an implicit solvation model, of an ab initio thermodynamics description that incorporates capacitive charging up to second order in the potential, as well as of a grand-canonical Monte Carlo sampling that explicitly evaluates configurational entropic effects in the adlayer. As a crucial insight, we observed an intricate error cancellation when several of these aspects were treated more approximately. A good agreement of a simulated CV with experimental data can thus not be taken uncritically as evidence that the employed level of theory was sufficient.

At the nominally best level of theory considered in this study (GC-MC sampling, implicit solvation energetics, and CHE+DL thermodynamics), we obtain a gratifying essentially quantitative agreement of the simulated CV with experimental reference data. The analysis provided suggests that this is the result of an appropriate description of key physics of this system, in particular, a coverage-dependent solvation stabilization due to a varying capability of the solvent to penetrate the adlayer and the disorder–order phase transition of the Br adlayer at higher coverages. Nevertheless, in view of the error cancellations observed at the lower levels of theory, this agreement should be scrutinized further in future work. Most prominently, we envision explicit electrolyte approaches as the next frontier that would provide the most valuable feedback on the true reliability of the implicit solvation method employed here employed implicit solvation method. Specifically, we hereby refer to both the parametrization of the implicit solvation model and its fundamental deficiencies in appropriately describing H-bonding networks and other directed solvent interactions at all. We consider the wealth of experimental CVs available for this system as an opportunity to systematically analyze such aspects with respect to a firm reference.

## Supporting Information Available

Digitalization of the experimental CVs, structure sampling, DFT calculations, mean-field theory, lattice grand-canonical Monte-Carlo, sensitivity analysis, order parameter and structure factor calculations, and more detailed data on the inclusion of double layer effects (PDF)
