# Supplementary material for: Ab Initio-Based Modeling of Thermodynamic Cyclic Voltammograms: A Benchmark Study on Ag(100) in Bromide Solutions
Source: J Chem Theory Comput. 2023 Dec 1;19(23):8815–25. doi: 10.1021/acs.jctc.3c00957 (PMC10720351; doi:10.1021/acs.jctc.3c00957)
Supplement: Supplementary file 1 — ct3c00957_si_001.pdf [file ct3c00957_si_001.pdf]

Supporting Information: *Ab-Initio*-Based Modeling of Thermodynamic Cyclic Voltammograms: A Benchmark Study on Ag(100) in Bromide Solutions

Nicolas Bergmann, Nicolas G. Hörmann,* and Karsten Reuter

Fritz-Haber-Institut der Max-Planck-Gesellschaft, Faradayweg 4-6, 14195 Berlin, Germany

E-mail: *hoermann@fhi-berlin.mpg.de

November 9, 2023

SI Experimental Cyclic Voltammograms

All CVs shown in Fig. 1 of the main text are from the echemdb database,[6] filtering the entries to match the experimental parameters of a Ag(100) working electrode and a Br-containing electrolyte, see Table S1 for details and references and Fig. S1 for a graphical display of the individual curves. The 10 resulting CVs were normalized to a common scan rate of $\nu = 50\,\mathrm{mV/s}$. The CVs from Refs. 1, 4, and 5, reportedly measured at $\nu = 10\,\mathrm{mV/s}$, deviate from the other CVs by a factor of exactly five, indicating an inconsistent labelling in the publications. Setting $\nu = 50\,\mathrm{mV/s}$ for these publications resulted in the expected behavior. To better compare the peak heights between different CVs, we ensured that the currents were centered around $j = 0\,\mu\mathrm{A/cm^2}$. For this, we used the

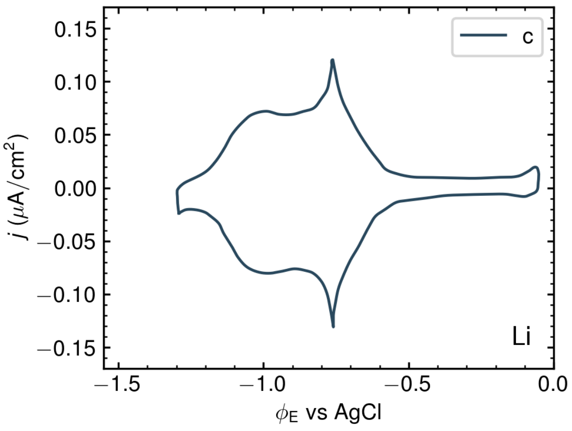

(a) with Li^{+}-counterion

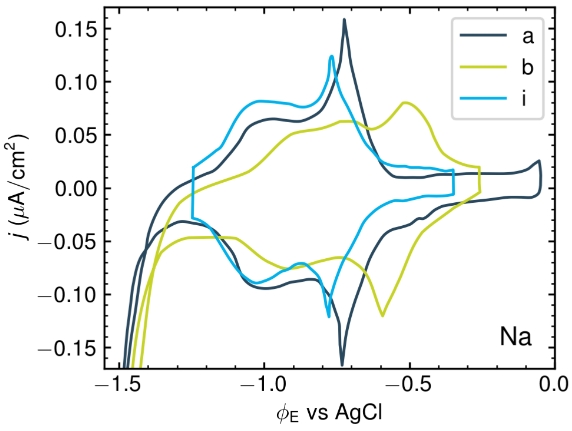

(b) with Na^{+}-counterion.

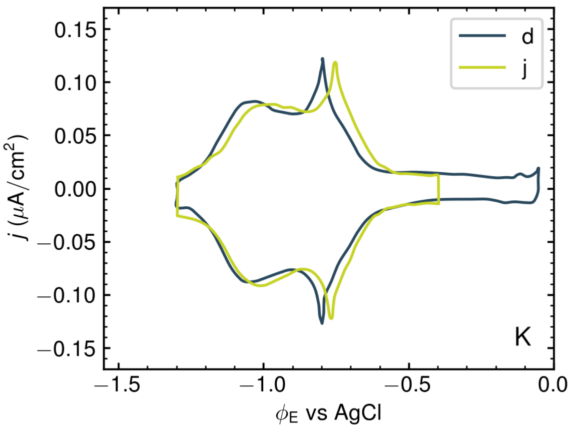

(c) with K^{+}-counterion.

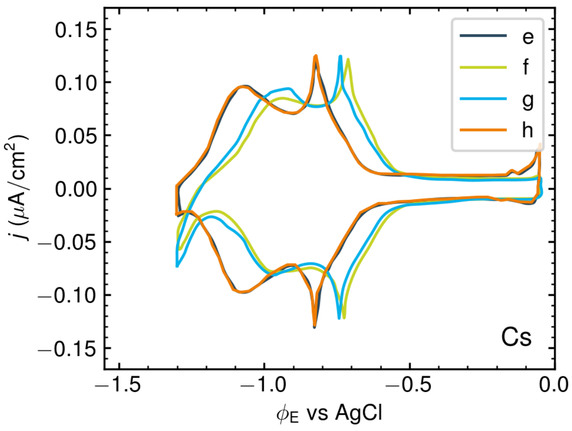

(d) with Cs^{+}-counterion.

Figure S1: Experimental CVs from the echemdb database, labelling refers to Table S1.

Table S1: Experimental data for the CVs from the echemdb database.

Fig. S1 label	$\phi_{\mathrm{E,\ ref}}$	pH	Cation	c_{Br^-} (mM)	v (mV/s)	Citation
a	Ag/AgCl		Na	10.00	10	Fig. 1a in Ref. 1
b	Ag/AgCl		Na	0.05	10	Fig. 1b in Ref. 1
c	Ag/AgCl	12.5	Li	100.00	50	Fig. 1a, blue line in Ref. 2
d	Ag/AgCl	12.5	K	100.00	50	Fig. 1a, green line in Ref. 2
e	Ag/AgCl	12.5	Cs	100.00	50	Fig. 1a, red line in Ref. 2
f	Ag/AgCl		Cs	10.00	50	Fig. 1b, blue line in Ref. 2
g	Ag/AgCl		Cs	10.00	50	Fig. 1b, red line in Ref. 2
h	Ag/AgCl	12.5	Cs	100.00	50	Fig. 1a, inset in Ref. 3
i	Ag/AgCl		Na	50.00	10	Fig. 6a, solid line in Ref. 4
j	SCE		K	10.00	10	Fig. 1a, solid line in Ref. 5

capacitive currents at potentials above the disorder-order P2 peak, taking the average of the anodic and cathodic currents above this potential. The difference of this average to $0\,\mu\mathrm{A/cm^2}$ was then subtracted from the rest of the CV. In a next step, we then applied a Nernstian shift to set the CVs to consistent values of c_{Br^-} and T. After this, small peak shifts between the different curves remained due to higher-order potential effects and electrolyte effects like the different cations used in the experiments. For a better visual comparison we therefore finally additionally shifted the curves to matching values of the cathodic P2 peak. Fig. S2 shows the effect of the different normalization steps on the experimental CV curves.

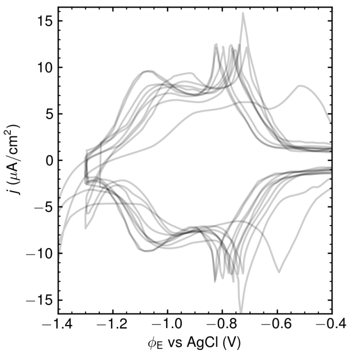

(a) CVs after normalization to the Ag/AgCl reference electrode.

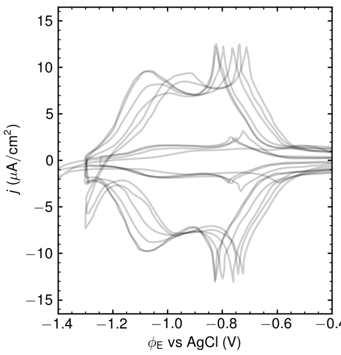

(b) CVs after correction to common ν =50 mV/s.

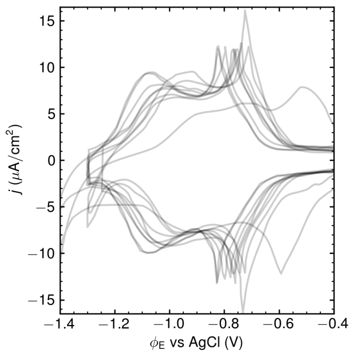

(c) CVs after baseline correction.

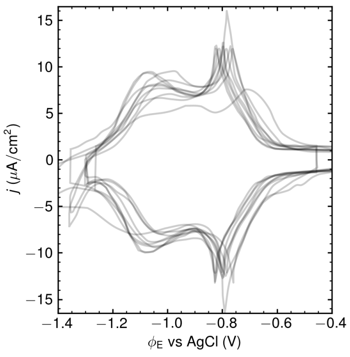

(d) CVs after $c_{\mathrm{Br}-}$ correction.

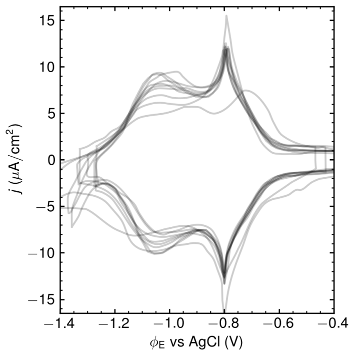

(e) CVs after final potential shift to match the cathodic sweep P2 peak.

Figure S2: Normalization steps applied to the experimental CVs from the echemdb database.

To get experimental values of the electrosorption valency l_{Br} and the total transferred electronic charge σ_{Br}, we first separated the anodic and cathodic currents of the normalized CVs. We then

subtracted the capacitive double layer baseline current, which we estimated from the essentially constant current at high potentials above $-0.3\,\mathrm{V\,vs\,AgCl}$. σ_{Br} was finally obtained by integrating the CV over the potential range from -1.3 to -0.4 V vs AgCl, as indicated by the red shaded area in Fig. 1 in the main text. For the electrosorption valency l_{Br}, σ_{Br} was divided by the ideal $\sigma_{\mathrm{Br,\ ideal}}$ of a 0.5 ML covered surface ($A_{\mathrm{site}} = 8.497\,\mathrm{\mathring{A}}^2$) with nominal $l_{\mathrm{Br}} = -1$.

$$l_{\mathrm{Br}} = \frac{\sigma_{\mathrm{Br}}}{\sigma_{\mathrm{Br,\ ideal}}} = \frac{\sigma_{\mathrm{Br}}}{\frac{-1\,\mathrm{e/ads}\times 0.5\,\mathrm{ads/site}}{8.497\,\mathrm{\mathring{A}}^2/\mathrm{site}}} = \frac{\sigma_{\mathrm{Br}}}{-94.268\,\mu\mathrm{C/cm^2}} \tag{S1}$$

Table S2: Integrated experimental data for the CVs from the echemdb database. The cathodic sweep direction of the CVs from Ref.[1] appears as an outlier and they were correspondingly not considered in the determined average values.

Fig S1 label	Citation	$\sigma_{\mathrm{Br}}^{\mathrm{Anodic}}$ $\mu\mathrm{C/cm^2}$	$l_{\mathrm{Br}}^{\mathrm{Anodic}}$	$\sigma_{\mathrm{Br}}^{\mathrm{Cathodic}}$ $\mu\mathrm{C/cm^2}$	$l_{\mathrm{Br}}^{\mathrm{Cathodic}}$
a	Fig. 1a in 1	60.658	0.643	102.256	1.085
b	Fig. 1b in 1	48.536	0.515	97.680	1.036
c	Fig. 1a, blue in 2	63.760	0.676	79.168	0.840
d	Fig. 1a, green in 2	64.097	0.680	74.643	0.792
e	Fig. 1a, red in 2	65.356	0.693	74.799	0.793
f	Fig. 1b, blue in 2	69.105	0.733	69.853	0.741
g	Fig. 1b, red in 2	73.581	0.780	68.650	0.728
h	Fig. 1a, inset in 3	64.812	0.687	74.468	0.790
i	Fig. 6a, solid in 4	65.862	0.699	78.042	0.828
j	Fig. 1a, solid in 5	65.511	0.695	71.607	0.760

SII Calculation of Chemical Potentials

SII.1 Density Functional Theory Calculations

SII.2 Ag and Br References

To determine a bulk chemical potential reference and an optimized lattice constant for Ag, we ran variable cell relaxations on the cubic Ag cell (Materials Project ID: 124). The Gamma-centered

k-grid was set to $(9 \times 9 \times 9)$. The bulk chemical potential was then determined via

$$\mu_{\mathrm{Ag}}^{\mathrm{bulk}} = \frac{1}{N_{\mathrm{Ag}}^{\mathrm{bulk}}} E_{\mathrm{Ag}}^{\mathrm{bulk}} \quad . \tag{S2}$$

Results for the PBE and revPBE exchange-correlation functionals are summarized in Table S3.

Table S3: DFT variable cell relaxation results for bulk Ag and for the PBE and revPBE exchange-correlation functionals. $d_{\mathrm{Ag-Ag}}$ is the relaxed Ag-Ag bond distance, $E_{\mathrm{Ag}}^{\mathrm{bulk}}$ is the relaxed DFT energy.

xc-functional	Formula	MP-id	$d_{\mathrm{Ag-Ag}}$	$E_{\mathrm{Ag}}^{\mathrm{bulk}}$	$\mu_{\mathrm{Ag}}^{\mathrm{bulk}}$
PBE	Ag_4	mp-124	2.915 Å	−16088.650 eV	−4022.162 eV
revPBE	Ag_4	mp-124	2.949 Å	−16090.987 eV	−4022.747 eV

For the Br chemical potential, we relaxed a Br_2-dimer in the center of a vacuum cell of size (10 Å × 10 Å × 10 Å). The Gamma-centered k-grid was set to $(1 \times 1 \times 1)$. Vibrational corrections were calculated with the Atomic Simulation Environment (ASE) Vibrations package (displacement magnitude=0.01 Å) and evaluated with the ASE IdealGasThermo package. Results for the PBE and revPBE exchange-correlation functionals are summarized in Tables S4 and S5.

Table S4: DFT relaxation results for the Br_2 gas-phase dimer and for the PBE and revPBE exchange-correlation functional. $d_{\mathrm{Br-Br}}$ is the relaxed Br-Br bond distance, $E_{\mathrm{Br}}^{\mathrm{dimer}}$ is the relaxed DFT energy.

xc-functional	$d_{\mathrm{Br-Br}}$	$E_{\mathrm{Br}}^{\mathrm{dimer}}$	$E_{\mathrm{Br}}^{\mathrm{dimer}}/2$
PBE	2.311 Å	−1274.390 eV	−637.195 eV
revPBE	2.311 Å	−1275.956 eV	−637.978 eV

Table S5: Vibrational energy terms for the Br_2 gas-phase dimer at $T = 298.15\,\mathrm{K}$ and $p = 1 \times 10^5\,\mathrm{Pa}$.

xc-functional	E_{ZPE}	H	TS	$(H - TS)/2$
PBE	0.019 eV	0.120 eV	0.759 eV	−0.320 eV

SII.3 Structure Sampling for Br adsorption

For Br adsorption on a 4×4 Ag(100) cell we consider three possible high-symmetry sites: top, hollow, and bridge. We run DFT relaxation calculations for all three possible sites, finding that for the $p(4 \times 4)$ adsorption pattern the hollow site is $\approx 150\,\mathrm{meV}$ more stable than the other sites, matching results previously reported[7]. Thus in the following, we only consider the fourfold hollow site as a possible adsorption site for Br.

To sample all relevant Br-hollow adsorption within the 4×4 Ag(100) cell, we find all possible occupation configurations, where no two neighboring sites are adsorbed. We then separate these into symmetrically unique configurations, counting the instances. The results are shown in the figure below, Fig. S3.

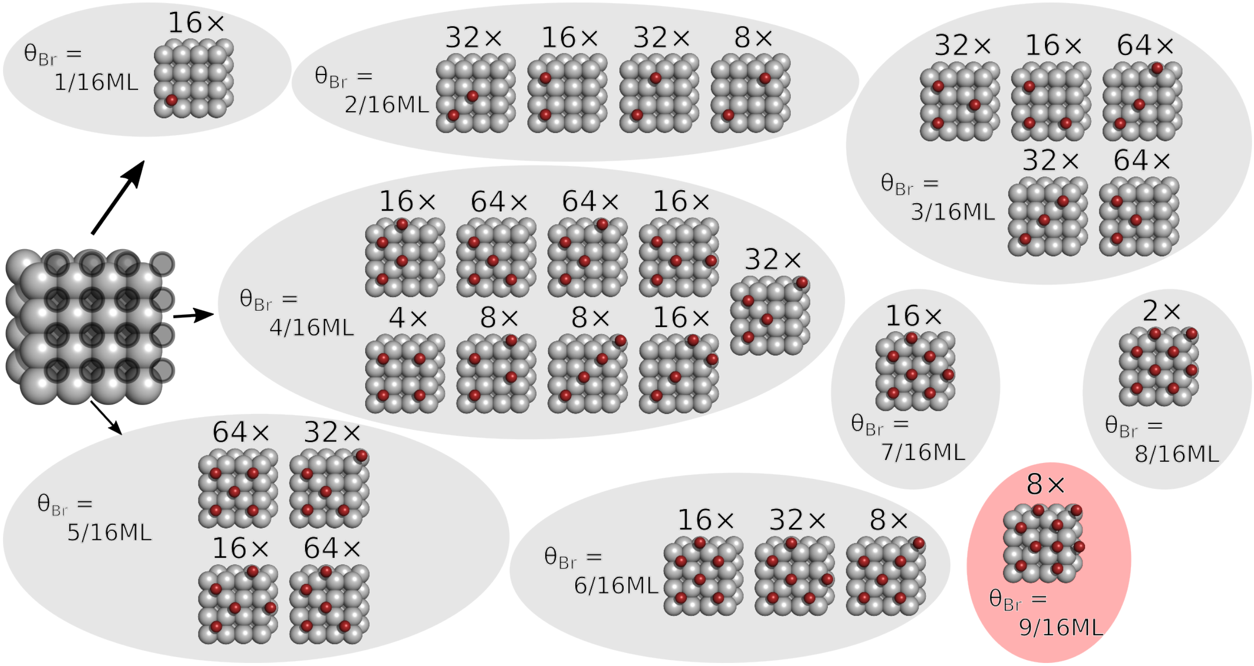

Figure S3: Sampled configurations of the 4×4 Ag(100) cell, only considering the fourfold hollow sites and not permitting nearest-neighbor occupations for coverages below 8/16 ML. The $X\times$ refers to the population of the specific configuration, which we take into account in the mean-field theory weighting.

SII.4 Adsorbed Br Vibrations

Vibrational corrections for adsorbed Br species on Ag(100) were calculated with the Atomic Simulations Environment (ASE) Vibrations package (displacement magnitude=0.01 Å) and evaluated with the ASE HarmonicThermo package.

Table S6: PBE vibrational energy terms for adsorbed Br on Ag(100) at $T = 298.15\,\mathrm{K}$

	Environment	N_{Br}	E_{ZPE}	U	TS	$(U-TS)/N_{\mathrm{Br}}$
$p(4\times4)$	Vacuum	1	0.013 eV	0.078 eV	0.171 eV	−0.093 eV
$c(2\times2)$	Vacuum	8	0.130 eV	0.630 eV	1.190 eV	−0.070 eV
$p(4\times4)$	Implicit Solv.	1	0.005 eV	0.078 eV	0.261 eV	−0.183 eV
$c(2\times2)$	Implicit Solv.	8	0.085 eV	0.625 eV	1.448 eV	−0.103 eV

SII.5 Weight-Averaged Density Functional Theory Results

Table S7 shows the population-weighted energies of our structural dataset. The values of $g_{\mathrm{exc}}^{\mathrm{CHE}}$ and $G_{\mathrm{ads}}^{\mathrm{CHE}}$ are used for both the lattice Grand-Canonical Monte Carlo (GC-MC) and mean-field theory (MFT) modeling approaches.

Table S7: Weight-averaged DFT results for Br on Ag(100). Adsorption and excess surface free energies are calculated for $\phi_{\mathrm{E}} = 3.83\,\mathrm{V}$ (the experimental PZC of Ag(100),[8–10]), $c_{\mathrm{Br}^-} = 0.1\,\mathrm{M}$, $T = 298.15\,\mathrm{K}$, and $\phi_{\mathrm{exp,Br}} = 5.53\,\mathrm{V}$. Coverages are in ML, all energies are in eV.

	Vacuum PBE			Vacuum revPBE			Implicit Solvent PBE		
θ_{Br}	E^{DFT}	$g_{\mathrm{exc}}^{\mathrm{CHE}}$	G_{ads}	E^{DFT}	$g_{\mathrm{exc}}^{\mathrm{CHE}}$	G_{ads}	E^{DFT}	$g_{\mathrm{exc}}^{\mathrm{CHE}}$	G_{ads}
0/16	-386113.721	0.433		-386172.087	0.362		-386115.243	0.386	
1/16	-387391.489	0.452	0.298	-387451.126	0.390	0.445	-387393.291	0.390	0.067
2/16	-388669.208	0.472	0.310	-388730.067	0.421	0.469	-388671.242	0.397	0.092
3/16	-389946.829	0.495	0.330	-390008.923	0.454	0.491	-389949.030	0.410	0.127
4/16	-391224.392	0.520	0.348	-391287.691	0.491	0.514	-391226.646	0.427	0.166
5/16	-392501.897	0.547	0.364	-392566.347	0.530	0.538	-392504.075	0.451	0.208
6/16	-393779.214	0.580	0.390	-393844.899	0.573	0.563	-393781.157	0.485	0.265
7/16	-395056.497	0.613	0.412	-395123.325	0.620	0.590	-395057.964	0.528	0.326
8/16	-396333.623	0.652	0.438	-396401.539	0.674	0.623	-396334.449	0.581	0.391
9/16	-397608.216	0.770	0.598	-397677.253	0.806	0.788	-397609.030	0.694	0.548

To calculate the coverage-dependent surface capacitance, we ran charged calculations via

modified Poisson-Boltzmann calculations for the clean surface and the 0.5 ML covered $c(2\times2)$ surface. The surface capacitance is the derivative of the charge with respect to the work function

$$\frac{1}{A}\frac{\mathrm{d}q}{\mathrm{d}\phi}\,\frac{[\mathrm{e}]}{[\mathrm{\mathring{A}}^2\mathrm{V}]} = C\frac{[1.602\times10^{-13}\,\mu\mathrm{C}]}{[1\times10^{-16}\,\mathrm{cm}^2\mathrm{V}]} = C\left[1.602\times10^{3}\,\mu\mathrm{F\,cm}^{-2}\right] \quad . \tag{S3}$$

Using the values in Table S9 and a cell surface area of 135.956 $\mathrm{\mathring{A}}^2$, we get 46.145 $\mu\mathrm{F\,cm}^{-2}$ for the clean surface and 28.385 $\mu\mathrm{F\,cm}^{-2}$ for the $c(2\times2)$ surface at the respective PZC. As has been shown in Ref. 11, the surface capacitance correlates linearly with θ_{Br}. We therefore set

$$C_0(\theta_{\mathrm{Br}}) = 46.145\,\frac{\mu\mathrm{F}}{\mathrm{cm}^2} - 35.520\,\frac{\mu\mathrm{F}}{\mathrm{cm}^2\mathrm{ML}}\,\theta_{\mathrm{Br}} \quad . \tag{S4}$$

Table S8: Work function values (in V) for Br on Ag(100). Additionally shown are the calculated work functions shifted to match the experimental PZC of the clean Ag(100) surface (3.83 V)[8–10].

	Vacuum		Implicit Solvent	
θ_{Br}	ϕ_0	$\phi_{0,\text{ corrected}}$	ϕ_0	$\phi_{0,\text{ corrected}}$
0/16	4.272	3.830	3.420	3.830
1/16	4.488	4.046	3.513	3.923
2/16	4.688	4.246	3.615	4.025
3/16	4.849	4.407	3.728	4.138
4/16	4.989	4.548	3.846	4.256
5/16	5.138	4.697	4.022	4.432
6/16	5.296	4.855	4.240	4.650
7/16	5.406	4.965	4.480	4.890
8/16	5.513	5.071	4.788	5.198
9/16	5.734	5.292	5.074	5.484

SIII Mean-Field Theory (MFT)

To run MFT-CHE simulations, we interpolated the discrete g_{exc} or G_{ads} values compiled in Table S7.

Table S9: Results of the charged calculations to determine the surface capacitances for the implicit solvent.

q (e)	$q\left(\frac{\mu C}{cm^2}\right)$	ϕ_{clean} (V)	$\phi_{c(2\times2)}$ (V)
-2	-47.138	2.833	
-1	-23.569	3.133	4.336
-0.5	-11.784	3.280	4.572
0	0.000	3.420	4.788
0.5	11.784	3.547	4.940
1	23.569	3.662	5.164
2	47.138	3.876	5.634
$C\left(\frac{\mu F}{cm^2}\right)$		46.145	28.385

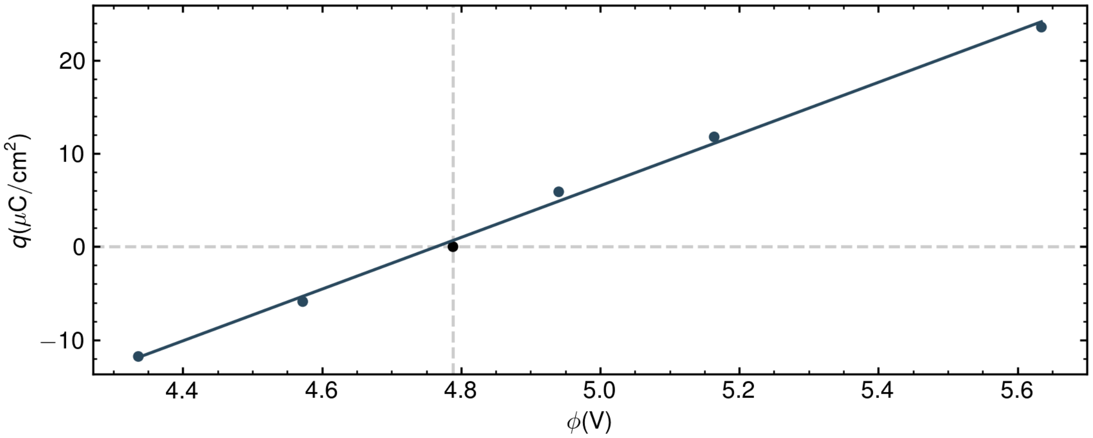

Figure S4: Charge-vs-potential relationship of the $c(2\times2)$ surface. Due to the very linear behavior of the curve, we can assume a potential-independent interfacial capacitance of $C_0^{(2\times2)} = 28.385\frac{\mu F}{cm^2}$.

To include the electrosorption valency in MFT, we use the expression from Ref. 11

$$l_{\mathrm{Br}}^{\mathrm{MFT}}(\theta_{\mathrm{Br}},\phi_{\mathrm{E}}) = \frac{q_{\mathrm{Br}}}{e} + \frac{A_{\mathrm{site}}}{e}\left(C_0^{\mathrm{MFT}}(\theta_{\mathrm{Br}})\frac{d\phi_0^{\mathrm{MFT}}}{d\theta_{\mathrm{Br}}} - \frac{dC_0^{\mathrm{MFT}}}{d\theta_{\mathrm{Br}}}\left(\phi_{\mathrm{E}} - \phi_0^{\mathrm{MFT}}(\theta_{\mathrm{Br}})\right)\right) \quad . \tag{S5}$$

In Fig. S5a, we reproduce the Ag(100)-Br CV for different interpolation methods also shown in Fig. 3 of the main text (second and third order polynomial interpolations (quadratic fit and cubic fit), cubic spline interpolation, and Gaussian process regression (GPR)). We additionally include the corresponding g_{exc} and the first $\partial g_{\mathrm{exc}}/\partial \phi_{\mathrm{E}}$ derivative. All interpolations were performed with the scikit-learn Python package[12]. For GPR, we used the sum of the white noise and squared exponential kernel as the kernel function. The quadratic and cubic fits, while very consistent with each other, do not remotely recreate the peak shape of the CV. This is due to the lack of dimensionality in the interpolation, which does not allow for multiple minima in the second derivative of the interpolation, as can clearly be seen in Fig. S5a. The cubic spline interpolation results in multiple peaks, corresponding to steep jumps in the coverage curve. These jumps seem to be small coverage increases in the low coverage limit, going up to coverages of around 0.25 ML. Then the jumps become larger in magnitude, either jumping first to around 0.4 ML, and then to 0.5ML, or directly to 0.5 ML.

The GPR results most faithfully resemble the experimental CV peak shape. Using GPR requires setting hyper-parameters though. For the employed kernels the significant hyper-parameters are the length scale l of the squared-exponential kernel, and, to a lesser extent, the noise level of the white noise kernel,

$$k\left(x_i,x_j\right) = -\exp\left(\frac{d\left(x_i,x_j\right)}{l^2}\right) \quad . \tag{S6}$$

While optimal values can be found with algorithms such as the BFGS algorithm, l's upper bound value (l_{max}) greatly affects the resulting CV, as shown in Fig. S5b. Small values of l_{max}, which is defined in units of θ, led to similar behavior as the cubic spline interpolation, introducing multiple small jumps in the coverage isotherm due to noise in the data. Increasing l_{max} to values above 0.45 ML, led to curves with only one CV feature at the peak onset, similar to the cubic polynomial

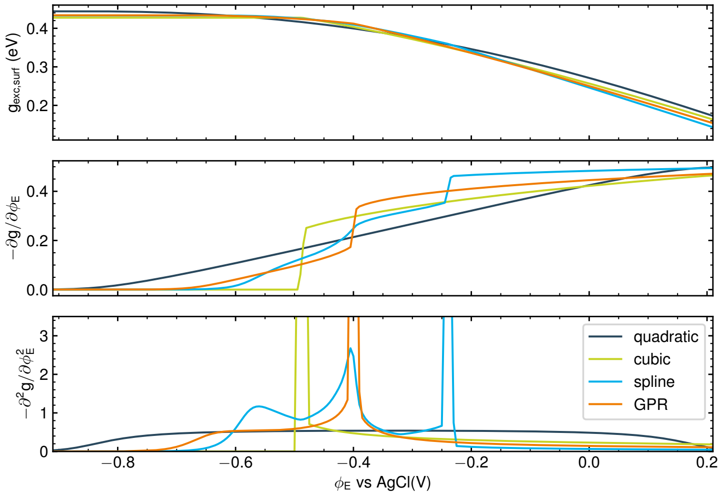

(a) Interpolated g_{exc}, as well as its first $\partial g_{\mathrm{exc}}/\partial\phi_{\mathrm{E}}$ and second $\partial^2 g_{\mathrm{exc}}/\partial\phi_{\mathrm{E}}^2$ derivatives for different interpolation methods (using vacuum energetics with CHE and MFT). The second derivative corresponds to the simulated CV.

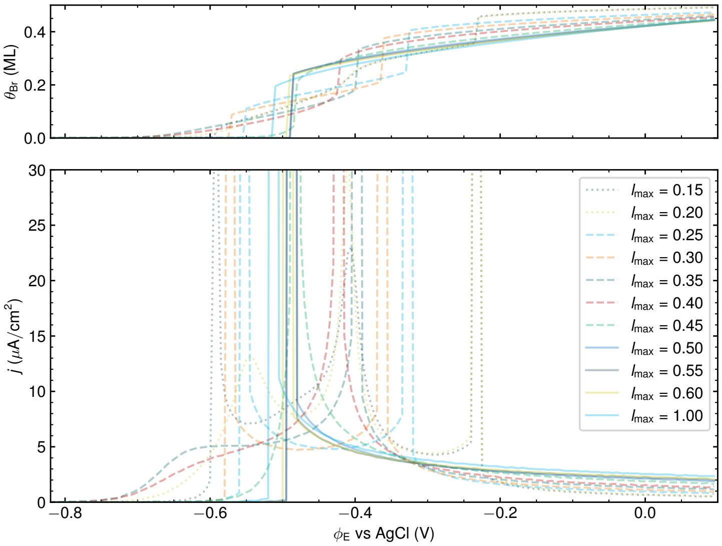

(b) Comparison of different maximum length scale bounds l_{max} value of the GPR kernel on the simulated CV using GPR-interpolated vacuum energetics with CHE and MFT.

Figure S5: Interpolation dependencies in mean-field theory.

interpolation. For $l_{\max}$ values between 0.25 and 0.45 ML, we observed two features.

SIV Lattice Grand-Canonical Monte Carlo simulations

The GC-MC simulations were run on a (18×18) 2D square lattice. Using the ICET Python package[13], we allowed diffusion moves via site swaps, as well as adsorption and desorption moves. At each potential, we ran 10^8 GC-MC trial steps and determined the thermodynamic Br-coverage by averaging over the last 10^5 MC steps.

SIV.1 Fitting the Cluster Expansion

Using the entire structural dataset consisting of the (4×4) clean slab, the 28 unique Br-adsorbate configurations, and one 9/16 ML surface to get potential nearest-neighbor interactions, we mapped the surfaces onto a two-dimensional grid, where an occupied hollow site is denoted by a "Br" atom and an unoccupied site by an "X" pseudoatom. To evaluate the thermodynamics at different applied potential conditions, we evaluated g_{exc} at intervals of 5 mV ϕ_{E}. Using the ICET python package, these energies and structures were fitted into a two-body cluster expansion (2b-CE)[13]. Figure S15 below summarizes the resulting potential-dependent onsite and 1st and 2nd NN interaction parameters.

SIV.2 Convergence Tests for the Monte-Carlo Simulations

Representative convergence tests were performed using the CHE thermodynamical description and energetics from vacuum DFT calculations. Four significant parameters were investigated: the cutoff distance of the 2b-CE, the number of MC steps, the probability rate of performing diffusion steps (so-called "swap rate"), and the cell size. For each of the plots in Fig. S6, the "default" parameters were: an initial surface without adsorbates, 10^8 total Monte Carlo steps with a swap rate of 0.75, a (12×12) surface, and a 2b-cutoff of 4.3 Å. After the first 90% of the total runtime, the final coverage was evaluated as the average of the final 10% of steps.

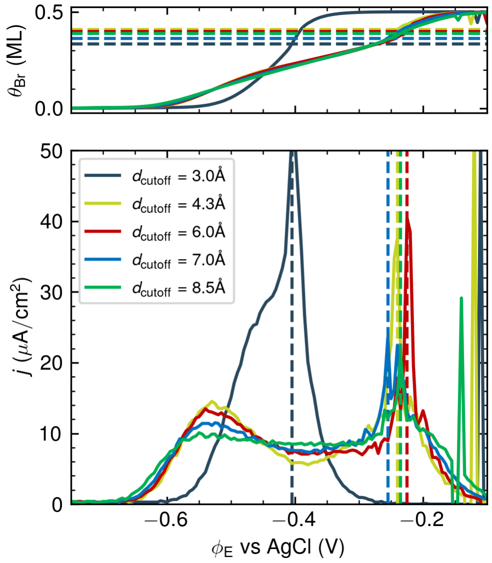

(a) Comparison of different Monte Carlo simulations for 2b-CEs with different cutoff distances (d_{cutoff}). The dashed lines indicate the position of the critical point of the disorder-order phase transition θ_c. For $d_{\text{cutoff}} = 3.0$ Å, which only considers the 1st-NN interaction, θ_c is at 0.35 ML. For all other cutoffs, $\theta_c \approx 0.39$ ML, agreeing with Ref. 14.

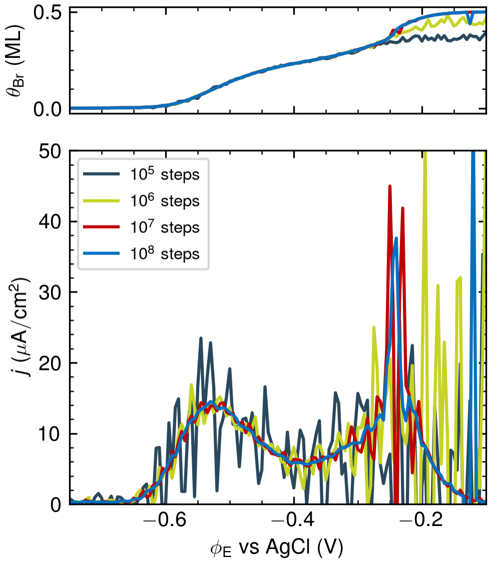

(b) Comparison of different Monte Carlo run lengths. Full convergence is reached for 10^8 steps.

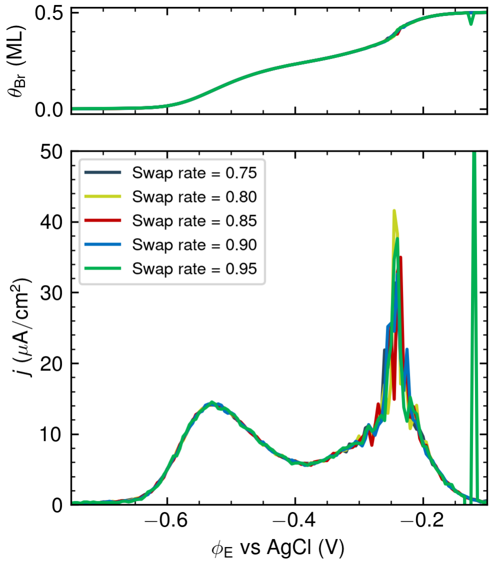

(c) Comparison of Monte Carlo simulations with a different "swap rate", referring to the probability that the Monte Carlo simulation attempts to swap two lattice sites, instead of an adsorption/desorption step.

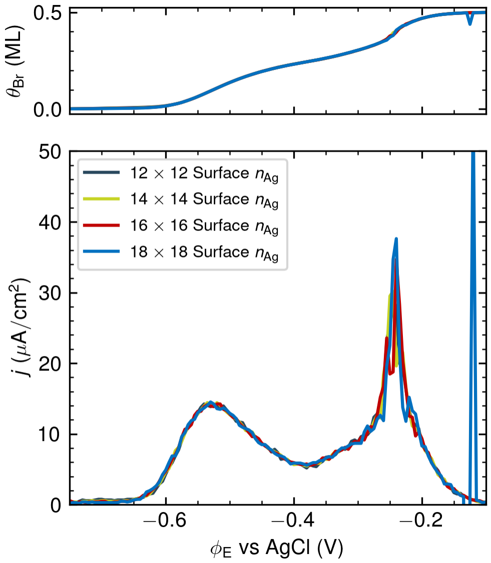

(d) Comparison of Monte Carlo simulations, using different cell sizes. Full convergence is reached at the employed (18×18) cell.

Figure S6: Checks performed for the lattice GC-MC simulations.

SIV.3 Determining the Electrosorption Valency with Grand-Canonical Monte-Carlo

Using CHE+DL thermodynamics within an IS model permits decoupling the electric potential from the adsorbate's chemical potential, which in turn allows determining non-integer values of l_{Br} via:[15]

$$l_{\mathrm{Br}} = -\frac{1}{e}\left(\frac{\partial \sigma_{\mathrm{Br}}}{\partial \theta_{\mathrm{Br}}}\right)_{\phi_E} = -\frac{1}{e}\frac{\left(\frac{\partial \theta_{\mathrm{Br}}}{\partial \phi_{\mathrm{E}}}\right)_{\tilde{\mu}_{\mathrm{Br}^-}}}{\left(\frac{\partial \theta_{\mathrm{Br}}}{\partial \tilde{\mu}_{\mathrm{Br}^-}}\right)_{\phi_{\mathrm{E}}}} \quad . \tag{S7}$$

In practice we accomplished this by fitting 2b-CEs with CHE+DL thermodynamics whilst slightly changing $c\left(\mathrm{Br}^-\right)$ around our reference value (0.1 M in our case),

$$\begin{aligned} \mu - \mu_{\mathrm{ref}} &= k_{\mathrm{B}}T\log\left(\frac{c\left(\mathrm{Br}\right)}{c_{\mathrm{ref}}\left(\mathrm{Br}\right)}\right) \\ \mu - \mu_{\mathrm{ref}} &= \phi_{\mathrm{E}} - \phi_{\mathrm{E,ref}} \quad . \end{aligned} \tag{S8}$$

We then ran GC-MC simulations for each set of concentrations. Figure S7a shows the coverage isotherm at $c_{\mathrm{ref}}\left(\mathrm{Br}\right) = 0.1\,\mathrm{M}$ for a small potential range around $-0.49\,\mathrm{V}$ vs AgCl, compared to the coverage isotherms at $-0.49\,\mathrm{V}$ vs AgCl for a small concentration range around $c_{\mathrm{ref}}\left(\mathrm{Br}\right) = 0.1\,\mathrm{M}$. Through eq. (S7), the resulting slope difference around the reference value can be transformed into l_{Br}.

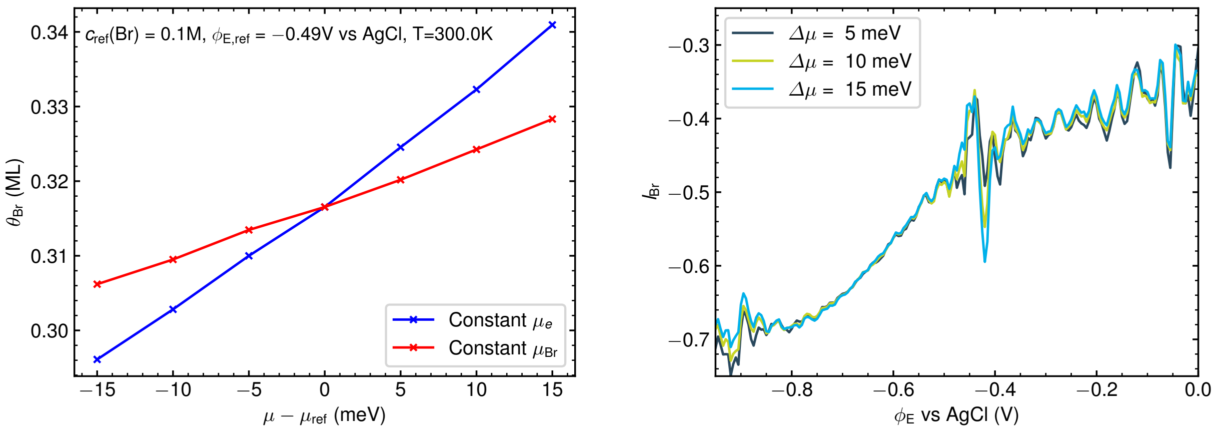

(a) Comparison of a coverage isotherm for a constant chemical potential over a small potential range and a coverage isotherm for constant applied electric potential over a small chemical potential range.

(b) Determining the electrosorption valency for different potential ranges. If $\Delta\mu$ is too large, a pronounced dip occurs at the disorder-order phase transition.

Figure S7: Determining l_{Br} within CHE+DL & implicit solvent GC-MC simulations.

SV Noise Bound Analysis

To analyze the sensitivity of the sampling methods, we added random noise to g_{exc} of the vacuum-CHE data at the indicated standard deviations σ_{noise}.

SV.1 Mean-field Theory

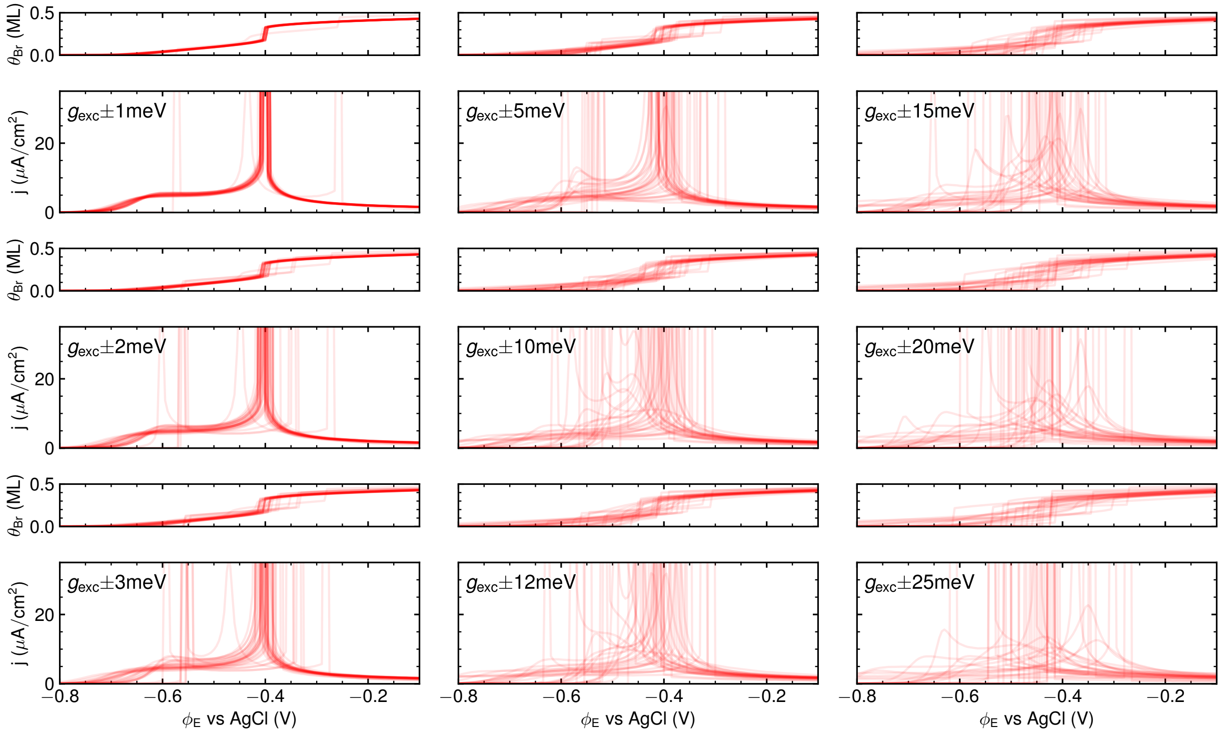

Figure S8: Results of GPR-interpolated CVs (using vacuum energetics and CHE) at different levels of white noise.

SV.2 Grand-Canonical Monte-Carlo

SV.2.1 Cluster Expansion Cutoff: 4.3 Å

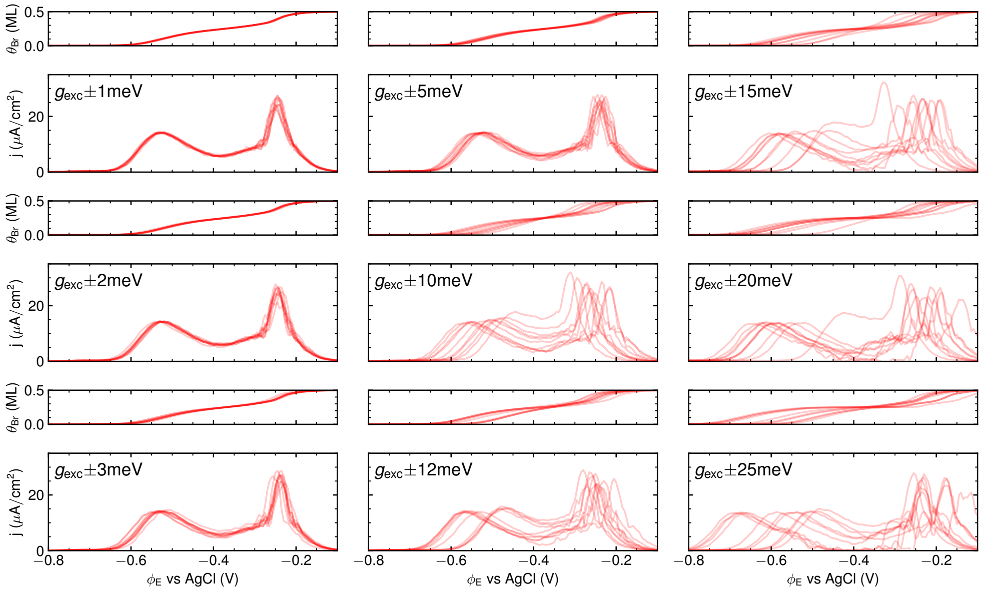

Figure S9: CHE & vacuum GC-MC CVs, using a 2b-CE with $d_{\text{cutoff}} = 4.3\,\text{Å}$, at different levels of white noise.

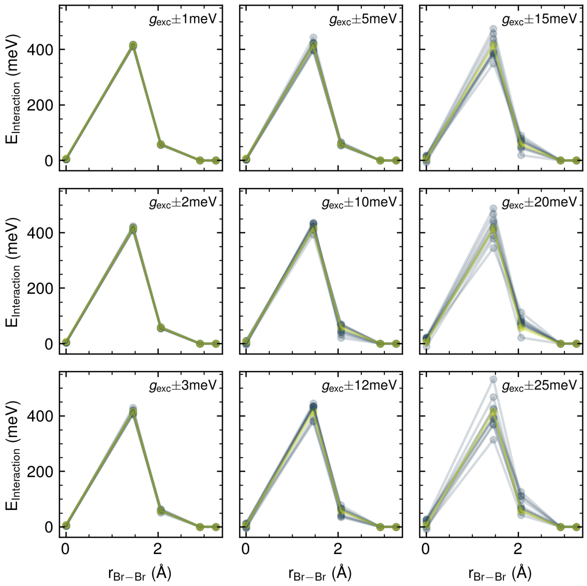

Figure S10: The2b-CE interaction energy terms at different levels of white noise for ($d_{\text{cutoff}} = 4.2\,\text{Å}$). The light-green line in each subplot corresponds to the unperturbed data.

SV.2.2 Cluster Expansion Cutoff: 7.0 Å

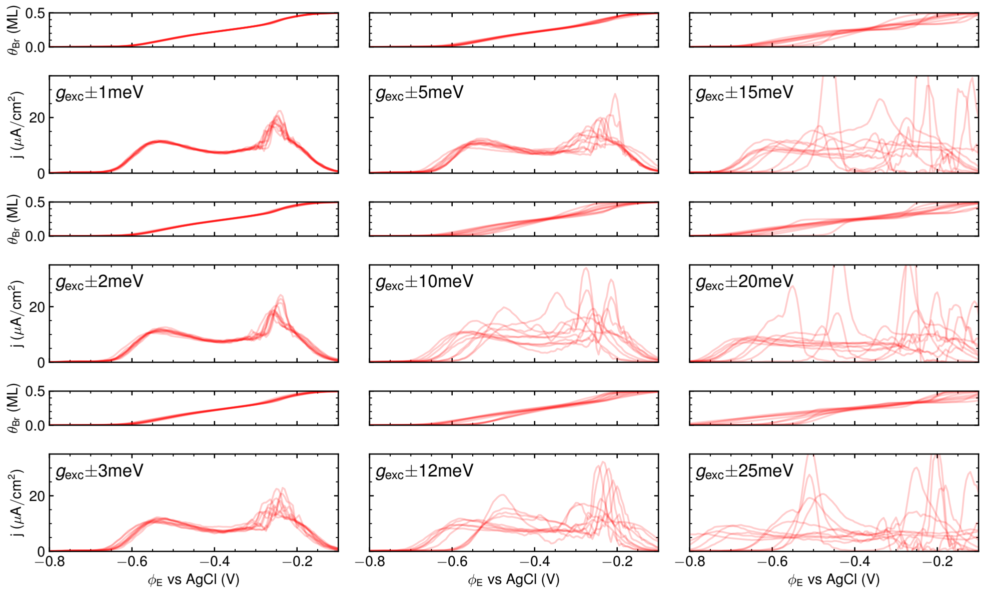

Figure S11: CHE & vacuum GC-MC CVs, using a 2b-CE with $d_{\text{cutoff}} = 7.0$ Å, at different levels of white noise.

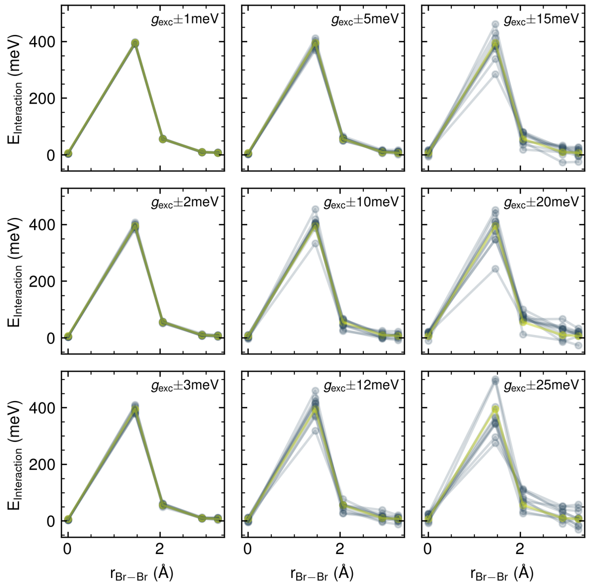

Figure S12: The 2b-CE interaction energy terms at different levels of white noise for ($d_{\text{cutoff}} = 7.0$ Å). The light-green line in each subplot corresponds to the unperturbed data.

SVI Determination of the Second Order Order-Disorder Transition

SVI.1 Order Parameters

The critical points of disorder-order transitions to periodic surface patterns can be found by defining order parameters Ψ for specific high-symmetry periodicities.[16,17] For the high-order symmetry $c(2\times2)$ phase, we employed the definition for $\Psi_{c(2\times2)}$ from Ref. 16. The critical electrode potential at which the transition occurs ($\phi_{\mathrm{E,C}}$) was defined as the point where Ψ becomes zero.

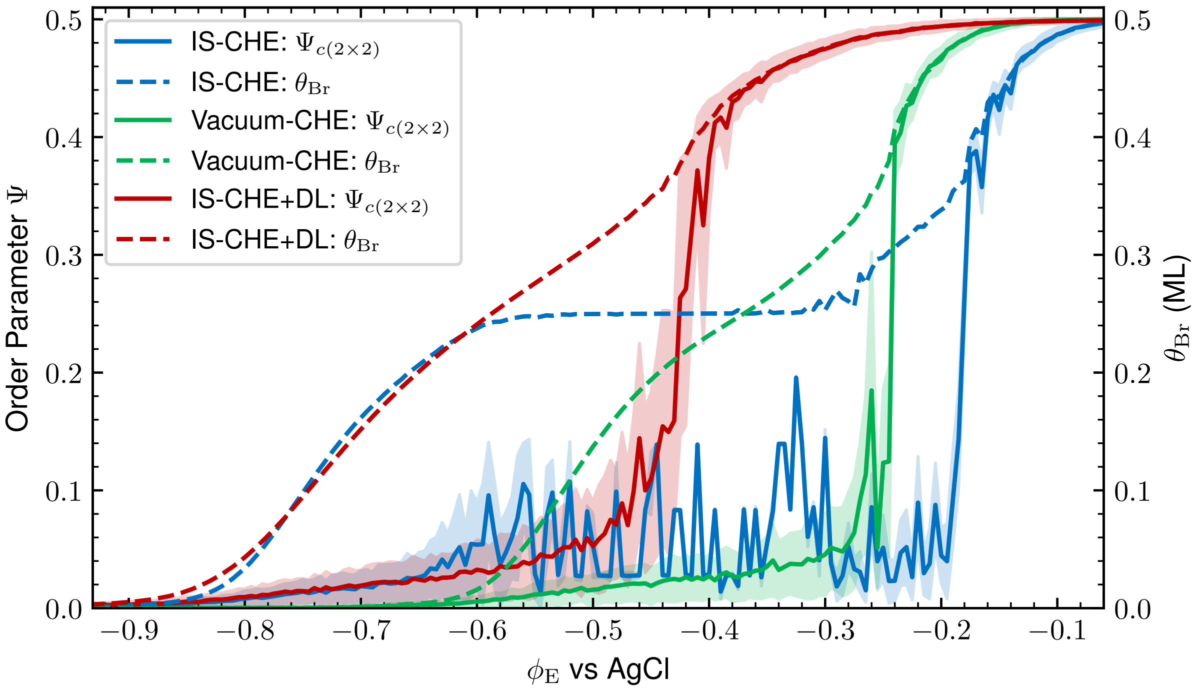

Figure S13: Order parameters for the $c(2\times2)$ surface pattern of all GCMC-CV curves examined in the main text

SVI.2 Structure Factor Evaluation

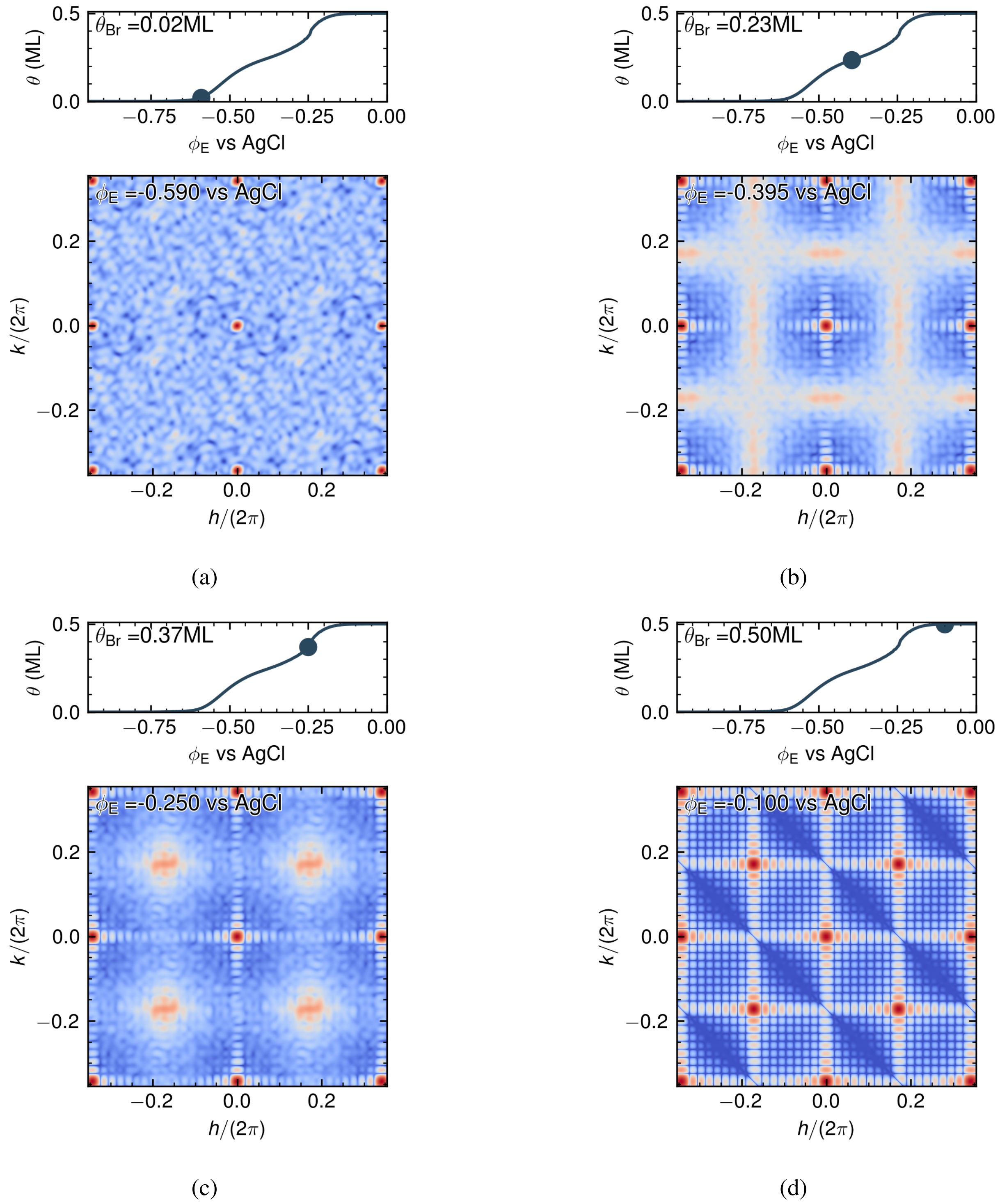

Figure S14: Structure Factors surrounding Br-environments at different potentials (marked by the dot in the coverage isotherm) and different coverages.

SVII Including Double Layer Effects

SVII.1 Model Effects on the Interactions within Cluster Expansions

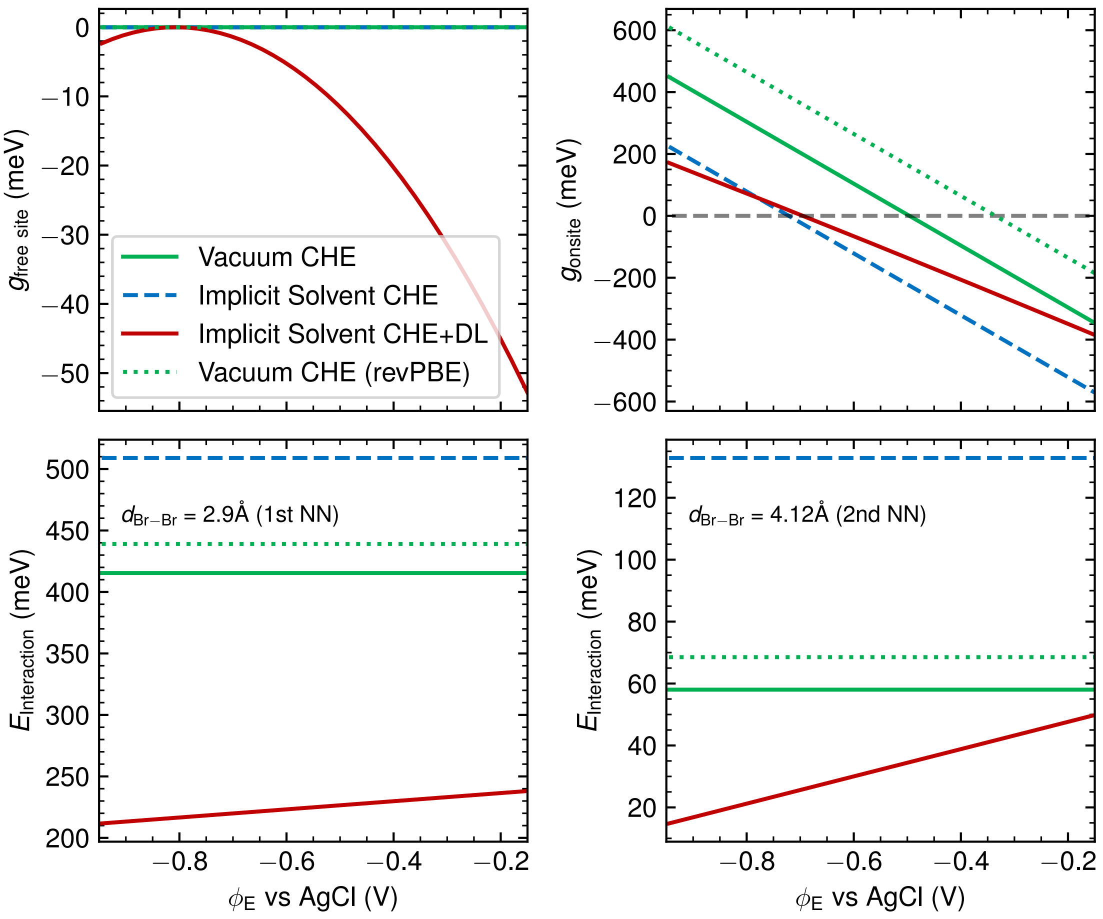

Figure S15: Evaluation of the energetic contributions in 2b-CEs ($d_{\text{cutoff}} = 4.3\,\text{Å}$) for the considered solvation and *ab initio* thermodynamic models.

SVII.2 The Electrosorption Valency: Mean-field Theory vs Grand-Canonical Monte-Carlo

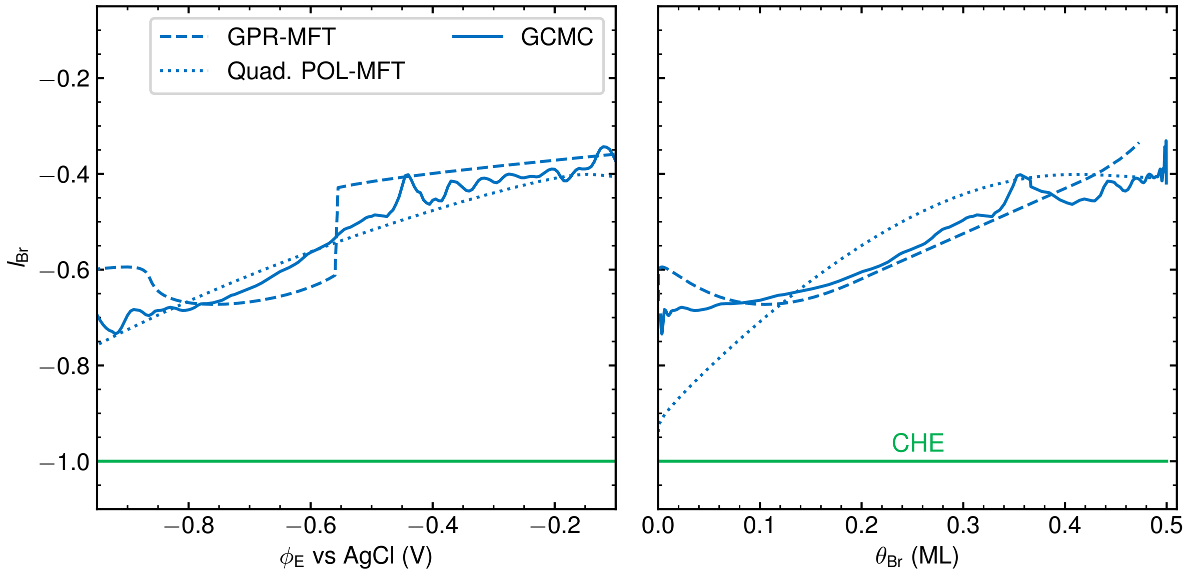

Figure S16: The electrosorption valency l_{Br} plotted against the electrode potential (left) and the Br coverage (right) for MFT and GC-MC.

References

(1) Endo, O.; Kiguchi, M.; Yokoyama, T.; Ito, M.; Ohta, T. In-situ X-ray absorption studies of bromine on the Ag(100) electrode. *Journal of Electroanalytical Chemistry* **1999**, *473*, 19–24, DOI: https://doi.org/10.1016/S0022-0728(99)00148-5.

(2) Nakamura, M.; Nakajima, Y.; Sato, N.; Hoshi, N.; Sakata, O. Structure of the electrical double layer on Ag(100): Promotive effect of cationic species on Br adlayer formation. *Physical Review B* **2011**, *84*, 165433, DOI: 10.1103/PhysRevB.84.165433.

(3) Nakamura, M.; Kaminaga, H.; Endo, O.; Tajiri, H.; Sakata, O.; Hoshi, N. Structural Dynamics of the Electrical Double Layer during Capacitive Charging/Discharging Processes. *The Journal of Physical Chemistry C* **2014**, *118*, 22136–22140, DOI: 10.1021/jp506979p.

(4) Ocko, B. M.; Wang, J. X.; Wandlowski, T. Bromide Adsorption on Ag(001): A Potential Induced Two-Dimensional Ising Order-Disorder Transition. *Physical Review Letters* **1997**, *79*, 1511–1514, DOI: 10.1103/PhysRevLett.79.1511.

(5) Wang, J. X.; Ocko, B. M.; Wandlowski, T. Lateral interaction energy derived from Frumkin isotherm for $c(2\times2)$ Br/Ag(100). **1997**,

(6) Engstfeld, A.; Rüth, J.; linuxrider; Hörmann, N. G. echemdb/echemdb: 0.6.0. 2023; `https://doi.org/10.5281/zenodo.7834993`.

(7) Wang, S.; Rikvold, P. A. Ab initio calculations for bromine adlayers on the Ag(100) and Au(100) surfaces: The $c(2\times2)$ structure. *Physical Review B* **2002**, *65*, 155406, DOI: 10.1103/PhysRevB.65.155406.

(8) Hörmann, N. G.; Andreussi, O.; Marzari, N. Grand canonical simulations of electrochemical interfaces in implicit solvation models. *The Journal of Chemical Physics* **2019**, *150*, 041730, DOI: 10.1063/1.5054580.

(9) Vayenas, C. G.; White, R. E.; Gamboa-Aldeco, M. E. *Modern Aspects of Electrochemistry*; Springer Science & Business Media, 2008; Vol. 42.

(10) Trasatti, S. Systematic trends in the crystal face specificity of interfacial parameters: The cases of Ag and Au. *Journal of Electroanalytical Chemistry* **1992**, *329*, 237–246, DOI: https://doi.org/10.1016/0022-0728(92)80219-T, An International Journal Devoted to all Aspects of Electrode Kinetics, Interfacial Structure, Properties of Electrolytes, Colloid and Biological Electrochemistry.

(11) Hörmann, N. G.; Reuter, K. Thermodynamic Cyclic Voltammograms Based on *Ab Initio* Calculations: Ag(111) in Halide-Containing Solutions. *Journal of Chemical Theory and Computation* **2021**, *17*, 1782–1794, DOI: 10.1021/acs.jctc.0c01166, PMID: 33606513.

(12) Pedregosa, F.; Varoquaux, G.; Gramfort, A.; Michel, V.; Thirion, B.; Grisel, O.; Blondel, M.; Prettenhofer, P.; Weiss, R.; Dubourg, V.; Vanderplas, J.; Passos, A.; Cournapeau, D.; Brucher, M.; Perrot, M.; Duchesnay, E. Scikit-learn: Machine Learning in Python. *Journal of Machine Learning Research* **2011**, *12*, 2825–2830.

(13) Ångqvist, M.; Muñoz, W. A.; Rahm, J. M.; Fransson, E.; Durniak, C.; Rozyczko, P.; Rod, T. H.; Erhart, P. ICET – A Python Library for Constructing and Sampling Alloy Cluster Expansions. *Advanced Theory and Simulations* **2019**, *2*, 1900015, DOI: https://doi.org/10.1002/adts.201900015.

(14) Taylor, D. E.; Williams, E. D.; Park, R. L.; Bartelt, N. C.; Einstein, T. L. Two-dimensional ordering of chlorine on Ag(100). *Physical Review B* **1985**, *32*, 4653–4659, DOI: 10.1103/PhysRevB.32.4653.

(15) Schultze, J.; Vetter, K. Experimental determination and interpretation of the electrosorption valency γ. *Journal of Electroanalytical Chemistry and Interfacial Electrochemistry* **1973**, *44*, 63–81, DOI: https://doi.org/10.1016/S0022-0728(73)80515-7.

(16) Zhang, Y.; Blum, V.; Reuter, K. Accuracy of first-principles lateral interactions: Oxygen at Pd(100). *Physical Review B* **2007**, *75*, 235406, DOI: 10.1103/PhysRevB.75.235406.

(17) Persson, B. Ordered structures and phase transitions in adsorbed layers. *Surface Science Reports* **1992**, *15*, 1–135, DOI: https://doi.org/10.1016/0167-5729(92)90012-Z.
